# Human Motion Enhancement via Tobit Kalman Filter-Assisted Autoencoder

**DOI:** 10.1109/access.2022.3157605

**Published:** 2022-03-08

**Authors:** NATE LANNAN, LE ZHOU, GUOLIANG FAN

**Affiliations:** School of Electrical and Computer Engineering, Oklahoma State University, Stillwater, OK 74078, USA

**Keywords:** Autoencoder, human motion manifold, depth sensors, motion capture, Tobit Kalman filter

## Abstract

We present a novel approach to enhance the quality of human motion data collected by low-cost depth sensors, namely D-Mocap, which suffers from low accuracy and poor stability due to occlusion, interference, and algorithmic limitations. Our approach takes advantage of a large set of high-quality and diverse Mocap data by learning a general motion manifold via the convolutional autoencoder. In addition, the Tobit Kalman filter (TKF) is used to capture the kinematics of each body joint and handle censored measurement distribution. The TKF is incorporated with the autoencoder via latent space optimization, maintaining adherence to the motion manifold while preserving the kinematic nature of the original motion data. Furthermore, due to the lack of an open source benchmark dataset for this research, we have developed an extension of the Berkeley Multimodal Human Action Database (MHAD) by generating D-Mocap data from RGB-D images. The newly extended MHAD dataset is skeleton-matched and time-synced to the corresponding Mocap data and is publicly available. Along with simulated D-Mocap data generated from the CMU Mocap dataset and our self-collected D-Mocap dataset, the proposed algorithm is thoroughly evaluated and compared with different settings. Experimental results show that our approach can improve the accuracy of joint positions and angles as well as skeletal bone lengths by over 50%.

## I. INTRODUCTION

**FIGURE 1.**
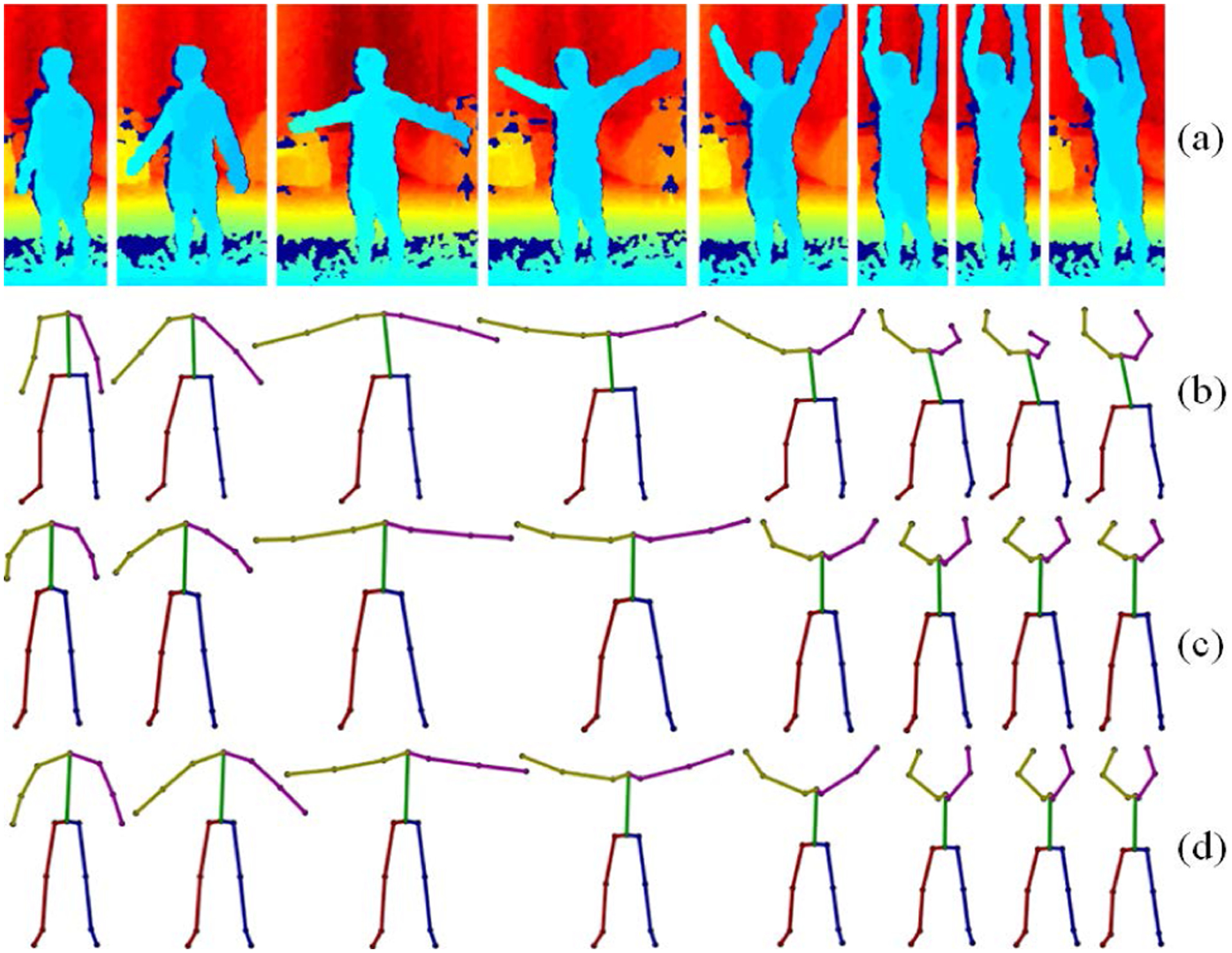
(a) A depth sequence from the MHAD dataset [42]; (b) D-Mocap data generated from (a) by using OpenPose [11]; (c) enhanced D-Mocap data by the proposed algorithm; (d) the ground-truth Mocap reference.

Marker-based optical motion capture (Mocap) technology is the gold standard for human motion data collection due to its high accuracy and low latency [5], [9], [26], [48]. However, there is a need for a practical low-cost solution in motion capture that provides reasonable accuracy and high flexibility. Previous research has yielded some promise with the use of inertial measurement units (IMUs) [8], but these devices suffer from drift and must be attached to the subject’s body. During the past decade, low cost RGB-D depth sensors have emerged as a promising alternative for motion capture (D-Mocap). They have proven useful in the clinical setting for gait assessment [17], [25], [40], [46], rehabilitation [47], human mobility analysis [32], and exercise systems [14]. In addition, there is a wealth of off-the-shelf or open source software which generates D-Mocap (Fig. 1).

Due to depth sensors’ limited range [7], interference susceptibility [38], and tendency toward self-occlusion errors [15], when compared to Mocap data, D-Mocap is low-quality and less accurate (Fig. 1(b)). Our aim is to combine two paradigms of human motion enhancement, filtering techniques and deep learning to achieve an affordable and practical markerless motion capture tool which alleviates the deficiencies of D-Mocap and could be used in a free-setting for a variety of biomedical and clinical applications (Fig. 1(c)).

Our method consists of two main elements, *learning* and *filtering*. The first involves a convolutional autoencoder based on [22], [23] which is trained on a large quantity of wide-ranging high-quality Mocap data. We chose a convolutional autoencoder to tackle the high dimensional nonlinear problem of human motion enhancement because of the ability of autoencoders to reduce the dimensionality of the data and extract noise through the use of a learned human motion manifold. The second involves the Tobit Kalman filter (TKF) which is a nonlinear Kalman filter devised to mitigate censored measurements in a temporal data sequence [2]. We adaptively vary the censoring limits of the TKF using the velocities of human joints in light of the fact that most errors in D-Mocap are accompanied by an unnatural shift in joint velocity [61]. Joints with a higher velocity component are more prone to error and joints that have been occluded have a higher velocity component caused by tracking failure. In [22] Holden *et al*. introduced manifold-based human motion synthesis via constraint-guided latent space optimization. The compelling idea of optimization in the latent space inspired us to develop a method in which a TKF filtered motion sequence with additional kinematic constraints could be used as a target for optimization. In this manner, we could capitalize on a separate method of motion enhancement and meld the two in the latent space of the autoencoder.

**FIGURE 2.**
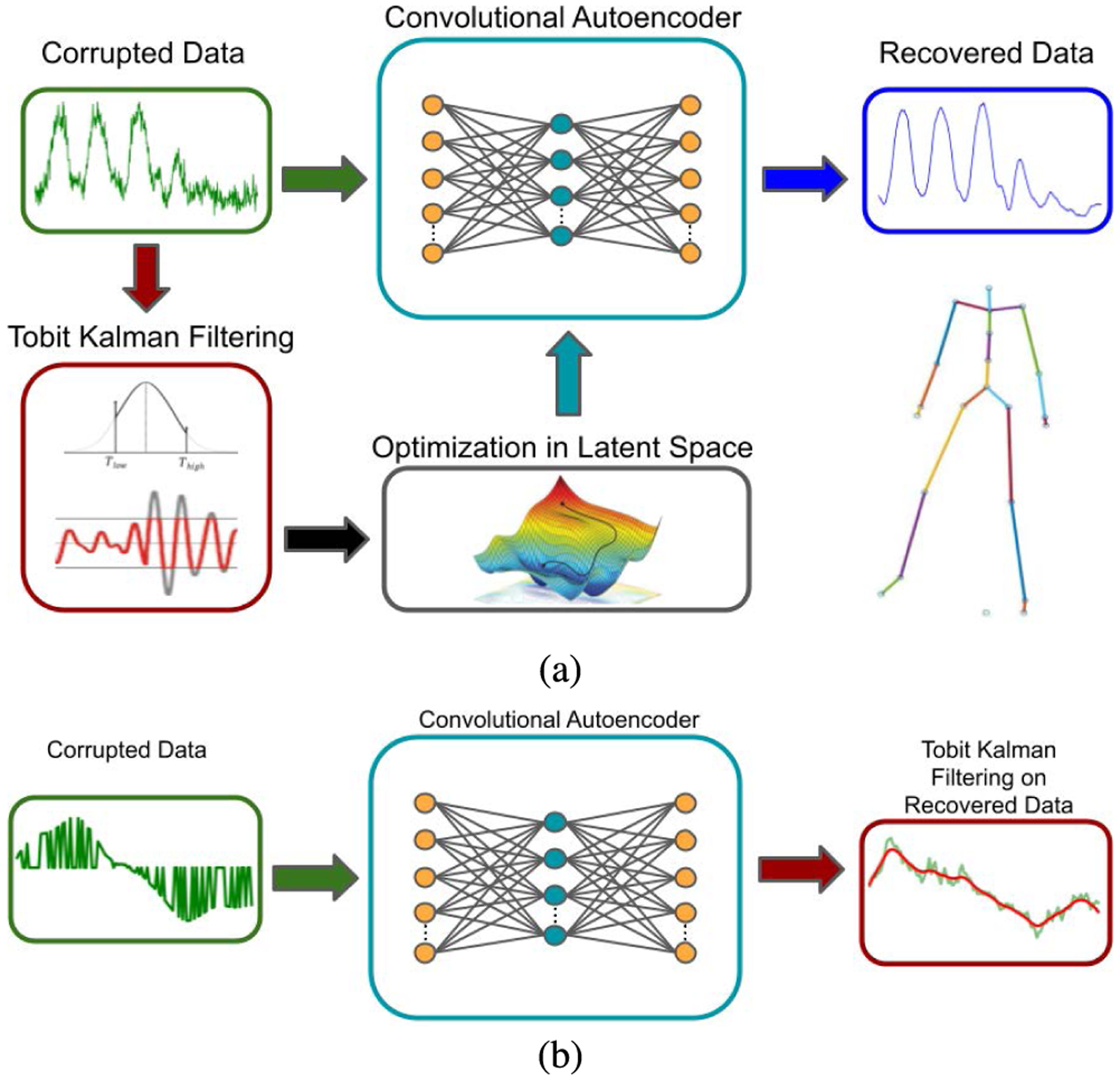
(a) The first paradigm: TKF-assisted autoencoder for motion denoising. (b) The second paradigm: TKF-refined autoencoder for missing data completion.

In our early work [29] we developed a method that applies Kalman filters after the use of the autoencoder and feeds the result back to the latent space for optimization. hlHowever, our recent studies reveal that while the autoencoder has the ability to project high-dimensional data onto a low-dimensional manifold, it may not preserve the natural kinematics of each joint trajectory along time due to its dimensionality reduction effect. This realization led us to an improved version [30] where the filter is applied in parallel with the autoencoder and the filtering output is directly used as the target for latent space optimization, resulting in more kinematics-compliant motion data enhancement, a new paradigm we call *TKF-assisted autoencoder* (Fig. 2(a)). As an alternative, we cascade the TKF with the autoencoder in a serial form to handle missing or lost motion data, leading to a second paradigm for motion enhancement called *TKF-refined autoencoder* (Fig. 2(b)). Both algorithms are evaluated quantitatively and comprehensively in this paper.

Although there are many recent approaches proposed for human motion enhancement or motion denoising, a common benchmark dataset for performance evaluation and algorithm comparison is lacking. In order to promote related research activities in the community, we have developed a new open source D-Mocap dataset that is extended from the Berkeley Multimodal Human Action Database (MHAD) [42] and available on GitHub.^1^ Our extended MHAD dataset includes a rich set of D-Mocap data generated from the RGB-D images which are time-synchronized and skeleton-matched with the optical Mocap reference data. Moreover, we captured our own D-Mocap data focused on human gaits by using a depth sensor side-by-side an optical Mocap system in two labs at Oklahoma State University (OSU). We have also generated a set of simulated D-Mocap data from the Carnegie Mellon University Motion Capture Database (CMU) [56] by considering two types of data corruption, i.e., additive white Gaussian noise (AWGN) and data drop-out (missing data). For the first time, our two algorithms (Fig. 2) are thoroughly evaluated against the three aforementioned D-Mocap databases with promising results.

To summarize, this manuscript provides new in-depth analysis of our TKF-assisted autoencoder through several previously unexplored means. These contributions are as follows:

- The proposed TKF-assisted autoencoder takes advantage of both joint-level kinematics and skeleton-level joint spatio-temporal modeling.
- We develop an extended MHAD dataset which includes D-Mocap data for use in human motion enhancement.^1^
- We provide comprehensive error analysis for simulated and real-world D-Mocap data that includes joint angles, bone lengths in addition to the traditionally used joint positions.

## II. RELATED WORK

In examining related work in human motion enhancement, we have focused our synopsis on the improvement of D-Mocap data. We have categorized these approaches into three groups, *filtering methods*, *machine learning methods*, and *deep learning methods*.

### A. FILTERING METHODS

Of all the traditional filtering methods, the Kalman filter (KF) has garnered the most attention in human motion enhancement. The linear KF was used in [55] constrained by joint dynamics in an effort to keep bone length constant. The authors improve upon the KF process through the use of the dynamics of human motion, but due to the nonlinear nature of D-Mocap data many researchers resorted to nonlinear KFs for motion denoising like the extended Kalman filter (EKF) or the unscented Kalman filter (UKF) [31], [49], [50]. Especially, the TKF may be best suited to deal with the nonlinearity and non-Gaussianity of D-Mocap due to the censored nature of occluded data [1]. Errors caused by self-occlusion were found to be reduced significantly more using the TKF over the KF in [36]. Using this knowledge Loumponias *et al*. then refined their work through a method for adapting the censor limits of the Tobit model showing that the troublesome aspects of D-Mocap can be confronted through the use of the TKF [37].

Traditional filtering methods have also been combined with evolutionary algorithms to varying degrees of success. In [54] a multi-objective genetic algorithm is combined with a particle filter to constrain bone length while filtering human motion data. Similarly in [16] a differential evolutionary algorithm (DE) is used in conjunction with the KF to maintain consistent bone length. Finally, DE is combined with the TKF in [61] to counteract changing bone lengths while benefiting from dynamically coupling the censoring limits of the TKF to joint velocity.

### B. MACHINE LEARNING METHODS

Machine learning methods in recent human motion enhancement research include dimensionality reduction, sparse coding, Gaussian Process (GP) models, and deep learning. In dimensionality reduction, authors in [51] utilize Greedy Kernel PCA [20] to represent human motion in the Hilbert space. This method can then be used to remove aspects of the data that are uncharacteristic of human motion. In sparse coding, researchers in [19], [58] used sparse coding dictionaries to reduce noise and outliers in human motion through *L*^1^ optimization. Likewise, a bone length and smoothing model is used with sparse coding dictionary learning to enhance human motion data. The work in [35] learns a mixture of Gaussian Processes and constrains velocity variations in optimization. Similarly, researchers in [14] train a GP regression model to map Kinect SDK data to Mocap data that has been captured simultaneously.

### C. DEEP LEARNING METHODS

By far, the most popular method in machine learning with regard to human motion enhancement is based on deep learning. The work in [43] uses two recurrent neural networks to improve D-Mocap data. These networks were each trained on two aspects of the kinematics of human motion, namely joint positions and joint velocities and the networks were interconnected. Sharing a similar spirit with our work, this work is an attempt to integrate the natural kinematics of the motion data into the neural network. Many researchers are focused on learning a lower dimensional representation of human motion data in the form of a manifold so that deviant motion can be removed from data [10], [22], [23], [29], [30], [33], [34], [57]. The authors in [10] employ three types of temporal encoders in an attempt to expunge unnatural motion. The work in [57] uses a network broken into three parts, a temporal section, a spacial section, and a residual section. The temporal section is a bi-directional LSTM encoder/decoder to learn time dependencies in human motion, the spacial section is a fully connected encoder/decoder that breaks up the human body into sections and learns spacial dependencies, and the residual network is a fully connected network which learns to remove high frequency noise. Researchers use a convolutional autoencoder in [22], [23] which simultaneously learns temporal and positional interdependencies of joints in order to build a robust manifold of human motion. The authors in [33], [34] expand on the ideas of [22] and incorporate a bidirectional long short-term memory (LSTM) network in a denoising autoencoder. This network is trained to reduce four loss functions, reproduction loss, perceptual loss, smoothness loss, and bone length loss.

The work by Li *et al*. [33], [34]recognizes an aspect of the work by Holden *et al*. [22] that we recognized in our own work, which is that much of the original natural kinematics of the data is lost when the convolutional autoencoder is used. This is because the autoencoder is trained on a rich dataset of varied Mocap data to create a manifold of human motion, but this manifold does not take into consideration the kinematics inherent amongst the sequential data. Li *et al*. use the addition of an LSTM to learn the natural kinematic dependencies of the original motion, whereas we have chosen to use the TKF to create a set of target motions that the data can be optimized toward in the latent space while adhering to the manifold learned by the convolutional autoencoder [29], [30]. Our TKF-assisted autoencoder builds upon the state of the art by combining the robust manifold obtained through deep learning and the preserved kinematics of human motion through recursive filtering. It uses filtering and deep learning methods and combines them in a synergistic way yielding a method that is greater than the sum of its parts.

## III. PROPOSED METHOD

**FIGURE 3.**
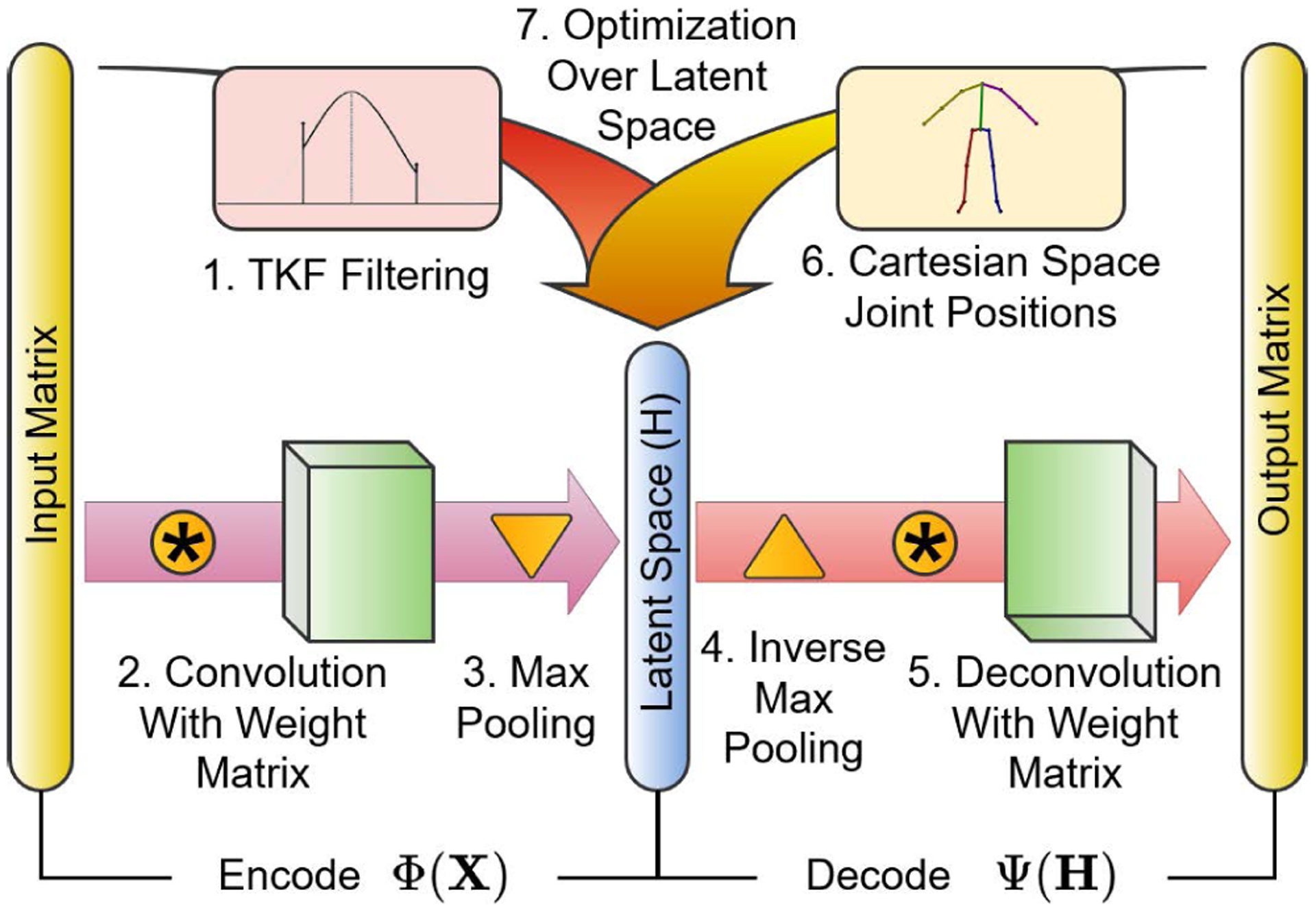
The illustration of the convolutional autoencoder where the TKF filtering output is projected into the motion manifold via latent space optimization in a 7 step enhancement process. 1) Original data are filtered with the TKF to produce a target matrix. 2) Original data are convolved with weight matrix and 3) maxpooled to form the latent space. 4) The latent space is inversely maxpooled and 5) deconvolved with the weight matrix to produce an estimate. 6) The estimate along with the target matrix form a cost function that is 7) optimized by modifying values in the latent space.

Our research goal is to capitalize on the capabilities of the TKF with dynamically adapting censor limits while conforming to the learned human motion manifold of a robustly trained convolutional autoencoder. The first paradigm (TKF-assisted autoencoder as shown in Fig. 2(a)) retains the natural kinematics or the original data while benefiting from hours of valid Mocap data learned by the deep neural network. The second paradigm (TKF-refined autoencoder as shown in Fig. 2(b))) is for human motion data suffering from excessive amounts of missing information (data drop-out). In this scenario the autoencoder is used first in order to generate initial estimation and the TKF is further used to refine the motion data. This paradigm is used because the autoencoder is more aptly able to deal with missing data at the onset than the TKF. In the TKF-assisted autoencoder method, data is enhanced through a 7 step process as shown in Figure 3. The original data are filtered with the TKF to produce a target for optimization over the latent space. The original data are also processed through the autoencoder to produce an estimate of clean motion data. This estimate, in conjunction with the TKF target, are used in a cost function and this cost function is optimized by modifying values in the latent space of the autoencoder. In the following, we will present a few key technical components in detail with a focus on the TKF-assisted autoencoder as the TKF-refined autoencoder is relatively straightforward.

### A. FRAMEWORK

The backbone of our method is a convolutional autoencoder originally proposed in [23]. The advantage of using this network is that it encodes human motion data into a subspace (latent space) where it is represented in a concise form and then decoded to build a recreation of the original data. If it is trained on a large amount of diverse quality human motion data, the network can be used to remove characteristics of the data that do not adhere to the learned motions. In addition, the lower dimensional subspace can be directly accessed in the autoencoder through the latent space values, and optimization can be performed on data without diverting from the motion manifold. Here we examine the anatomy of the autoencoder which includes two sections, an encoder, Φ, and a decoder, Ψ (Fig. 3).

The backbone of our autoencoder is a convolutional neural network (CNN) instead of a fully connected or recursive network. A CNN was chosen over a fully connected or recursive network, due to the ability of the convolution step to learn associations of joints in space as well as time. This is because the encoder section of the autoencoder consists of a convolution of weights and biases, or convolutional filters, along the frames of motion of the input motion data, thereby simultaneously learning positional and temporal dependencies among joint position data. The decoder performs an inverse convolution and is trained to reconstruct the original data. This is done so that when data are introduced that contain elements that do not adhere to valid human motion, these elements are expunged as there is no representation in the latent space for them. As shown in Figure 3, the input data are filtered with a TKF in parallel to the autoencoder in order to create an optimization target. This target is compared to the Cartesian space output of the autoencoder, but all adjustments for optimization are done in the latent space to incorporate the kinematic benefits of the TKF without deviating from the motion manifold.

This neural network framework was coded in Python 3.9 with the Theano 1.0.5 Python library. The Theano library was used for all symbolic differentiation and optimization over the latent space of the convolutional autoencoder. The Tobit Kalman filtering process was coded in Matlab R2021b and the resultant data was converted to a NumPy array for use in optimization over the latent space of the autoencoder using Theano and Python.

### B. AUTOENCODER

**FIGURE 4.**
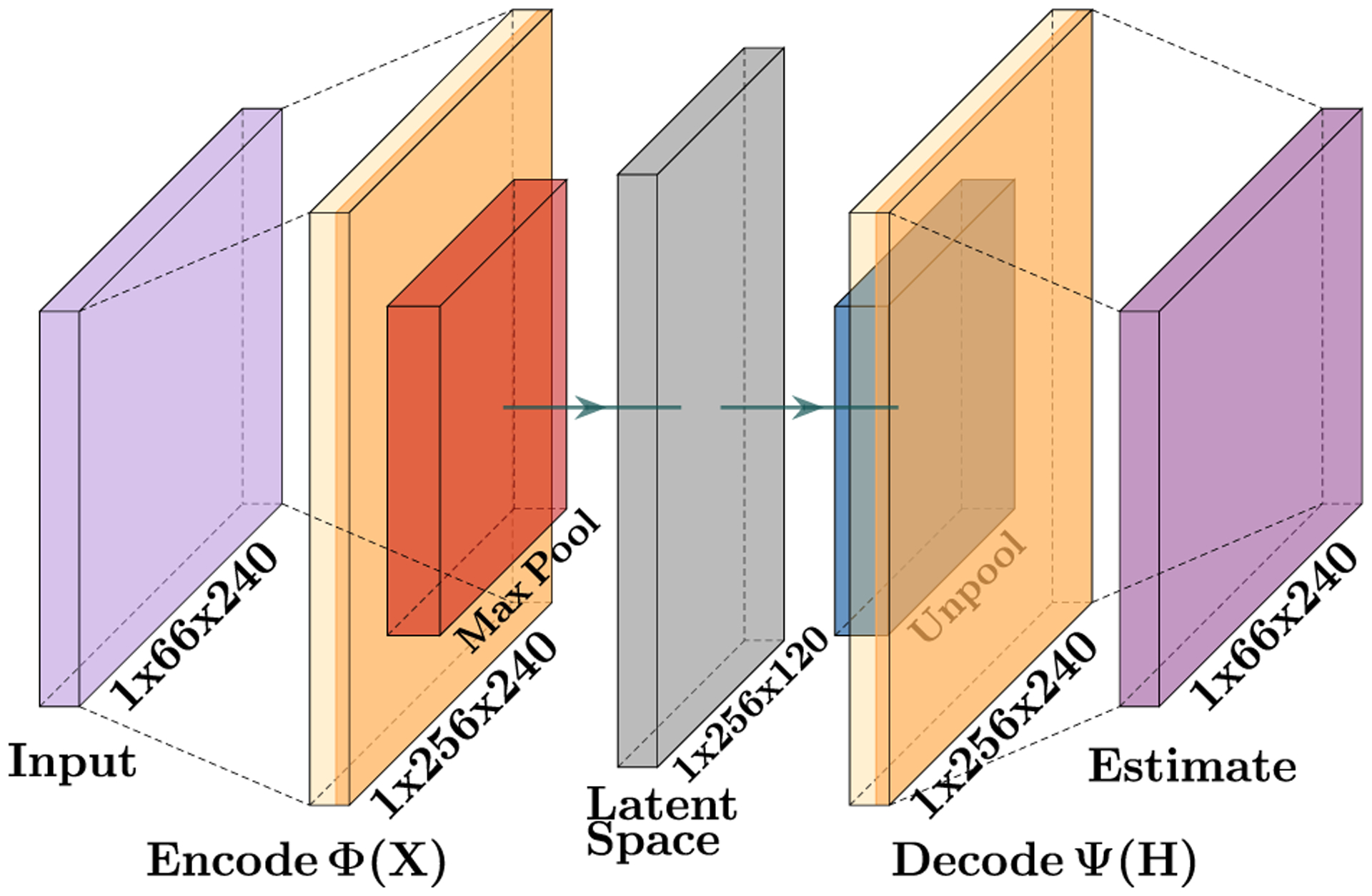
The matrix structure of convolutional autoencoder for a 22 joint skeleton over 240 frames.

The convolutional autoencoder processes data in the form of a matrix of joint positions in Cartesian space over frames of motion. In our experimentation we used data of varying skeletal model types and therefore the network was retrained for each of these models. Training clips were 240 frames which corresponds to four seconds of human motion. For a model with, *N*, joints each of which have 3 Cartesian dimensional values, training matrices were $X\in {\mathbb{R}}^{240\times 3N}$. Figure 4 depicts the autoencoder training on a 240-frame clip with a skeletal model of 22 joints. However, after training the autoencoder is not limited to 240-frame clips and can be arbitrarily long. Thus, the structure of the network is variable, where a general input matrix would be $X\in {\mathbb{R}}^{M\times 3N}$, where *M* is the number of frames of the input matrix.

The purpose of the encoder (Φ(**X**)) is to convert the joint positions of the human motion to the latent space through the convolutional filters. In this work there are 256 convolutional filters each 25 frames wide yielding a weight matrix of $W\in {\mathbb{R}}^{256\times 3N\times 25}$ and a bias vector of $b\in {\mathbb{R}}^{256}$. The weight matrix is convolved across the input with a stride of one frame, and the bias vector is added before a max pooling operation (*S*(·))is performed, removing the lowest value of each pair of values along the dimension of the frames of motion, effectively halving the size of the resultant matrix. The output of the maxpooling operation is activated with the rectified linear unit (*ReLU*(*x*) = *max*(*x*, 0)) yielding the latent space (**H**),

(1) H=Φ(X)=ReLU(S(X*W+b)).

Equation (1) is the mathematical process that the input matrix, **X**, undergoes in transformation into the latent space of the autoencoder.

The purpose of the decoder (Ψ(**H**)) is to transform the data in the latent space back into the Cartesian space using the inverse operations of the encoder. First, inverse maxpooling, *S*^−1^(**H**), is approximated since any value lost during max pooling is not recoverable. In this case, the max value saved is repeated in two adjacent units. The bias vector is subtracted and inverse convolution is performed on the result. This is, the process of convolving with $\tilde{W}$, the same weight matrix from the encoder only reflected across the frame axis, and transposed with regard to the other two axes. Thus, an approximation of the original input, $\hat{X}$,

(2) $$\hat{X}=\Psi (H)=\left(S^{-1}(H)-b\right)*\tilde{W},$$

is generated from the output of the decoder and is based on the information learned in the latent space. Equation (2) is the mathematical process that the latent space, **H**, undergoes in transformation into the input estimation, $\hat{X}$.

### C. TOBIT KALMAN FILTER FOR HUMAN KINEMATICS

The pivotal contribution that our work offers is the synergistic incorporation of the TKF with the learned motion manifold of the convolutional autoencoder. This is done through optimization over the latent space of the autoencoder using the TKF filtered data as a target. For our verification purposes, the TKF was used to assist the autoencoder for comparative analysis. This section focuses on the fundamental elements and principles of the TKF as they apply to human kinematics.

The KF is widely used for tracking applications because of its considerable efficiency and simple implementation. However, the standard KF is better suited for use in linear systems, while human motion is inherently non-linear. In contrast, the TKF is a novel approach that more accurately models human motion data obtained from D-Mocap systems. The TKF is a nonlinear KF based on the Tobit model, a statistical model which addresses data that has been censored in some way [53].

As with traditional KFs, the state value in the TKF is predicted at each time step *k* from the immediately preceding instant, by a state-transition model *F* and process noise. Hence, the state-transition function is expressed as

(3) $$X_{k}=FX_{k-1}+w_{k},w_{k}\sim N\left(0,Q_{k}\right)$$

where in Equation (3) the process noise *w*_*k*_ is assumed to be a multivariate Gaussian with zero mean and covariance *Q*_*k*_.

In human motion enhancement, we aim to estimate the movement of joints in Cartesian space; therefore, we assume the true state of the system is the positions, *p*, and velocities, *v*, of the joints

(4) $$X_{k}={\left[p_{k}^{\left(x\right)}p_{k}^{\left(y\right)}p_{k}^{\left(z\right)}v_{k}^{\left(x\right)}v_{k}^{\left(y\right)}v_{k}^{\left(z\right)}\right]}^{T}.$$

In Equation (4) the superscript denotes the three-dimensional directions of Cartesian coordinates, and this yields a state vector of 6 elements. Likewise, the observed measurements in human motion enhancement can be defined as the coordinates of skeletal joints, *q*,

(5) $$Y_{k}={\left[q_{k}^{\left(x\right)}q_{k}^{\left(y\right)}q_{k}^{\left(z\right)}\right]}^{T},$$

which correspond to the joint positions of the observed D-Mocap data. Given what we know of Newtonian physics, we can predict the state of the system using (4) and a constant velocity (CV) model [60],

(6) $$F=\left[\begin{array}{cccccc} 1 & 0 & 0 & \Delta t & 0 & 0\\ 0 & 1 & 0 & 0 & \Delta t & 0\\ 0 & 0 & 1 & 0 & 0 & \Delta t\\ 0 & 0 & 0 & 1 & 0 & 0\\ 0 & 0 & 0 & 0 & 1 & 0\\ 0 & 0 & 0 & 0 & 0 & 1 \end{array}\right],$$

where Δ*t* is the period of the sampling of human motion data. The original D-Mocap data are represented in the form of a dynamic skeleton that has a fixed number of moving joints. Therefore, we create a multi-channel motion sequence where each channel corresponds to the 3D trajectory of one joint, and the joint-level TKF is performed on each channel individually. In other words, data association in the TKF is based on the joint index in the skeleton. At each joint, we use the CV motion model in (6) as the transition matrix in (3) to model the joint-level kinematics for the TKF.

The Kalman filter is a model-based optimal filter, which requires exact knowledge of process and measurement models as well as process and measurement noise statistics. However, for some applications, such as target tracking [12], [18], the exact knowledge of the process model is difficult to derive. For these kinds of applications, the process model is based on the first principle of common physical laws. As reviewed in [24], the constant velocity (CV) model is a commonly used assumption to derive a process model. Due to the nature of human motion, the exact motion model is challenging to establish and derive. In [6] the authors attempted to remedy tracking error caused by rapid changes in target motion by fusing head-pose priors with a modified CV model. This modified model uses a windowed approach to smooth the velocity calculation and produce an instantaneous prior belief of where the target will move. This method attempts to address nonlinearities in human motion through a windowing of velocity information. In [36], the CV model was used in the TKF for human motion denoising where the velocity of the joint is updated frame by frame and that shows some promising results. Here, the nonlinearities of human motion are handled purely through the use of the TKF. Inspired by [36] and [6], we have chosen to use the CV model in the TKF with windowed velocity incorporated into dynamic threshold calculations of the TKF as the joint-level motion model in our research.

Since the observed measurements in (5) are the joint positions, the observation model merely extracts the position portion of the state and disregards velocity information,

(7) $$H=\left[\begin{array}{cccccc} 1 & 0 & 0 & 0 & 0 & 0\\ 0 & 1 & 0 & 0 & 0 & 0\\ 0 & 0 & 1 & 0 & 0 & 0 \end{array}\right].$$

In Equation (7), *H* assumes the positions are observed directly and is only subject to the noise associated with measurement devices.

The crux of the TKF is the use of the Tobit model with regard to measured data. In enhancing D-Mocap, the benefit of this model is based on the assumption that some joint positions may be occluded or blocked by other parts of the body during the movement, resulting in only a portion of the true state value being observed. Two thresholds are specified using the Tobit model, *TL*, and *TH* to censor values that cross these thresholds. Values that are below the lower threshold, *TL*, and values above the upper threshold, *TH*, instead take on the value of the respective threshold.

This is an important development in human motion enhancement as joint data are often partially obtained due to occlusion and algorithmic inadequacy. The TKF allows for the predictive ability of the KF while compensating for incomplete measurements assuming the thresholds are adaptable and chosen in an intelligent fashion. In our work these thresholds are dynamically adjusted using the previous joint position and the absolute value of the maximum velocity of the joint, |*v*_*max*_|, for a small window of frames centered at the previous time step. This window size ranges from 50 to 80 frames to include a complete human action, increasing the accuracy of our estimation, while minimizing computation expense. A different set of upper and lower thresholds are used for each Cartesian direction, so if we consider the *x* coordinate at time step *k* we have,

(8) $$TH_{k}^{\left(x\right)}=p_{k-1}^{\left(x\right)}+\left|v_{max}^{\left(x\right)}\right|\Delta t\;TL_{k}^{\left(x\right)}=p_{k-1}^{\left(x\right)}-\left|v_{max}^{\left(x\right)}\right|\Delta t,$$

where $p_{k-1}^{\left(x\right)}$ is the *x* coordinate of joint position *p* at the previous time step. More information on the thresholds selection in (8) can be found in [36]. With the introduction of thresholds into the KF, the linear relationship between the observation model in (7) and the state vector in (4) no longer exists. Therefore, we must introduce a new measurement model for our system,

(9) $$\rho_{k}^{\left(x\right)}=\{\begin{array}{ll} q_{k}^{\left(x\right)}, & TL_{k}^{\left(x\right)}<q_{k}^{\left(x\right)}<TH_{k}^{\left(x\right)}\\ TL_{k}^{\left(x\right)}, & q_{k}^{\left(x\right)}\leq TL_{k}^{\left(x\right)}\\ TH_{k}^{\left(x\right)}, & q_{k}^{\left(x\right)}\geq TH_{k}^{\left(x\right)}, \end{array}$$

where *ρ* is a latent variable that can be defined as a vector of all three Cartesian coordinates as ***ρ***. Equation (9) splits the observation into three regions, above the upper threshold, below the lower threshold, and between the thresholds. Due to this change, the Kalman gain in the update step becomes

(10) $$K_{k}=R_{XY_{k}}R_{YY_{k}}^{-1},$$

where $R_{XY_{k}}$ is the cross-covariance between state and error and $R_{YY_{k}}$ is the variance of the error. A detailed derivation for (10) can be found in [2]. In addition, the a posteriori estimate for the system state, *X*_*k*__|*k*_, is modified from the familiar linear KF by

(11) $$X_{k|k}=X_{k|k-1}+K_{k}\left(\rho_{k}-E\left(Y_{k}\right)\right),$$

where the expectation, *E*(*Y*_*k*_), is a combination of the expectations of all three sections of the measurement model in (9). Thus, the computation for this expectation is given by

(12) $$E\left(Y_{k}\right)=p_{k}^{\left(uc\right)}⊙\rho_{k}+p_{k}^{\left(l\right)}⊙TL_{k}+p_{k}^{\left(h\right)}⊙TH_{k},$$

where ⊙ is the Hadamard product and $p_{k}^{\left(uc\right)}$, $p_{k}^{\left(l\right)}$, and $p_{k}^{\left(h\right)}$ are the probability vectors for the three sections of the measurement model [62]. Details regarding these probability vectors and the derivation of (12) can be found in [2]. Equation (11) replaces the calculation for the updated state estimate in a traditional linear KF but still acts as a weighting between the a priori state estimate, *X*_*k*__|*k*−1_, and the observation using the Kalman gain. Finally, the a posteriori estimate of error covariance, *P*_*k*__|*k*_, is modified slightly from the linear KF by

(13) $$P_{k|k}=\left(I-K_{k}p_{k}^{\left(uc\right)}H\right)P_{k|k-1}.$$

Equation (13) differs only slightly from the traditional linear KF, taking into account the three-sectional nature of the motion model.

This TKF method is applied to the D-Mocap to generate a target for optimization in the latent space of the autoencoder. In this way the network can benefit from the preserved kinematics and adjustment for censored data rooted in the TKF.

### D. LATENT SPACE OPTIMIZATION

In our previous work, [29], we devised a method to optimize the output of the autoencoder over the latent space using target motion data. We originally filtered the joints of the approximation $\left(\hat{X}\right)$, to create our target for optimization. This method was used with moving average filters which do not consider the kinematic nature of the data. However, in introducing Kalman filtering [30], it is necessary to filter the original data and not the approximation to preserve the kinematic content so that it can add relevance to the autoencoder. The ability to successfully meld the TKF and a human motion manifold hinges on adherence to the manifold while optimizing toward the TKF. The capability to do this requires that we analyze our progress in Cartesian space while modifying the latent space. If we consider the approximation, $\hat{X}$, from the output of the decoder in (2), as a matrix of approximated joint positions, we can consider the individual joint positions, $\hat{p}$, for each joint, *j*, and each frame *k*. This separation would result in the general term, ${\hat{p}}_{k,j}$, for the coordinates of an approximated joint at a specific frame. With reference to (2), the inverse mapping

(14) $$H=\Psi^{-1}(\hat{X}).$$

yields the representation of the approximation in the latent space. Equation (14) provides a useful tool for mapping all of the output of the autoencoder back into the latent space, but to modify individual joints we use (15) to define the mapping of a particular joint at a specific frame, ${\hat{p}}_{k,j}$ into the latent space as

(15) $$p_{k,j}^{H}=\Psi^{-1}\left({\hat{p}}_{k,j}\right),$$

where $p_{k,j}^{H}$ represents the values in the latent space that produce the joint of interest. Using this definition, we can optimize the autoencoder output toward a Cartesian target as long as the target corresponds frame-by-frame to the approximation of the autoencoder. We define our target as a set of desired joint positions at each frame where *r*_*k*,*j*_ is a particular target joint at a specific frame. We then use *L*^2^ optimization on the difference of the autoencoder approximation with our target in Cartesian space

(16) $$\text{Cost}(H)=\sum_{j}\sum_{k}{\left\|\Psi \left(p_{k,j}^{H}\right)-r_{k,j}\right\|}_{2},$$

but modify only the values in the latent space to do so. The optimization in (16) is implemented using the Adam method for stochastic gradient descent in the Theano coding environment [52]. It is important to note that the TKF-refined autoencoder (Fig. 2(b)) was used for human motion with excessive missing data. In this case, the TKF has difficulty mending large voids in data and dealing with transient changes. Therefore, the autoencoder was used first to create an approximation since the manifold is better equipped to deal with this type of data.

## IV. TRAINING METHODS

**FIGURE 5.**
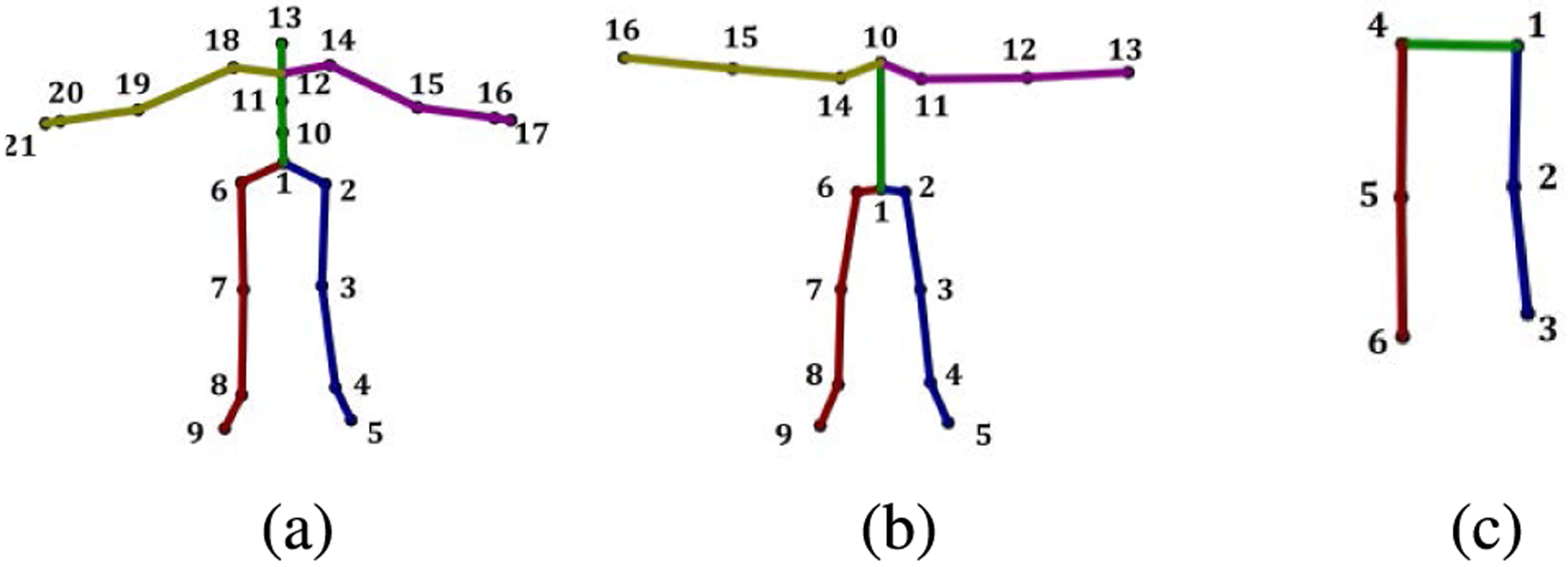
Skeletal structures of the three testing datasets used for quantitative analysis. The autoencoder was retrained with these three structures by omitting unneeded joints from the simplified CMU structure. (a) Simplified 21 joint CMU model. (b) Extended MHAD model. (c) OSU model.

**FIGURE 6.**
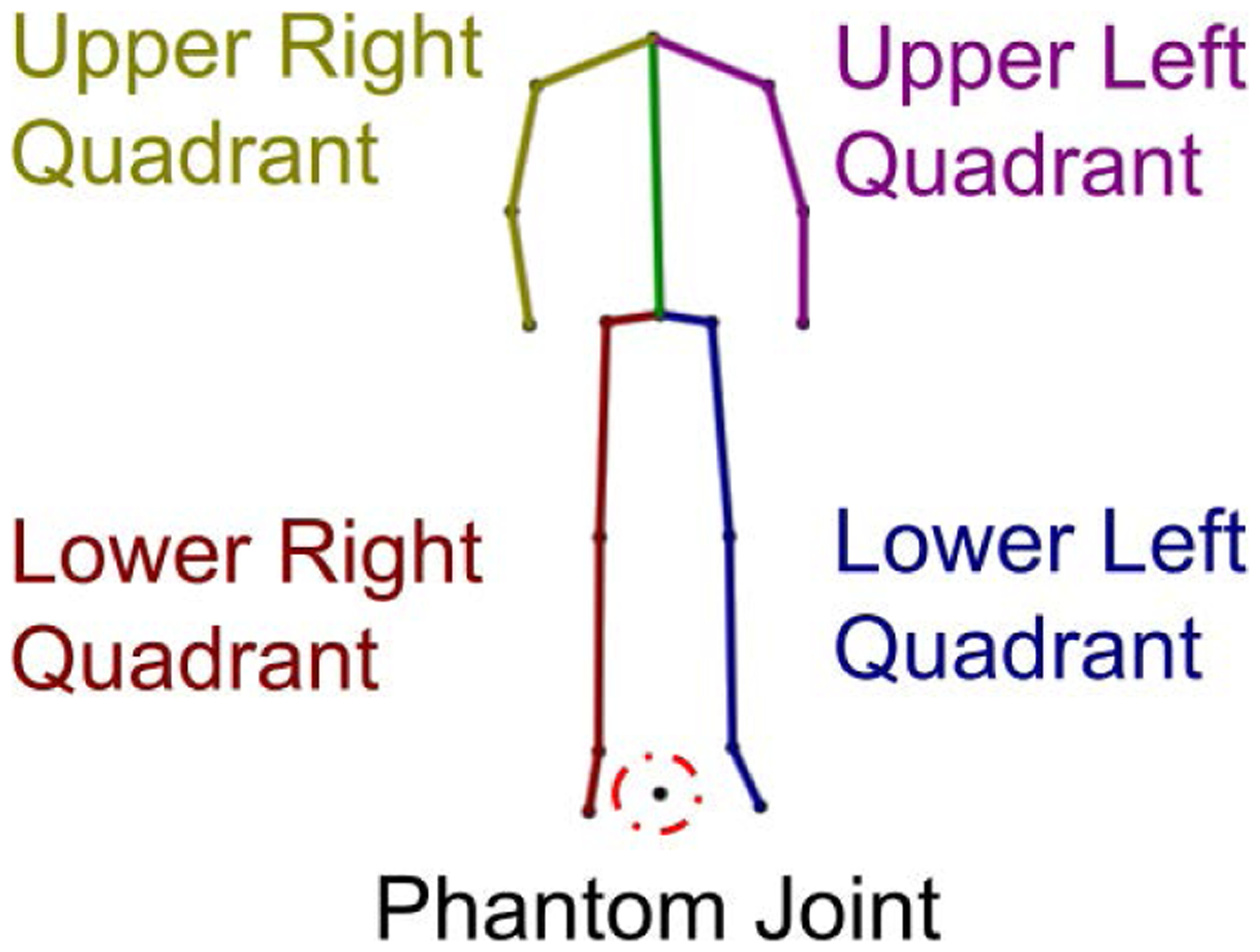
Four quadrants of the skeletal structure with phantom joint projected from the center of the hips to the *y* = 0 plane to promote learning of local body motion.

The training data for the network consisted of four datasets of diverse accurate Mocap data, namely the CMU dataset, the MHAD dataset, the Mocap Database HDM05 [41], and the data originally collected in [22], [23]. In order to maintain a consistent skeletal structure, all data were retargeted to a single homogeneous structure consistent with the CMU data set with inverse kinematics on matching joint angles. This process involved first, creating a target skeleton based on a simplified 21 joint CMU skeleton (Fig. 5(a)). This target skeletal structure ensures the training data have constant bone lengths, size, and structure. Second, corresponding joint angles from the dataset source skeleton were copied to the target skeleton. Third, the dataset source skeleton was scaled to match the size of the target skeleton. Fourth, full-body inverse kinematics was used to match the joint positions of the target skeleton to those of the dataset skeleton while maintaining constant skeletal structure. Details regarding this process can be found in [59]. Finally, joint angles were used to convert the data to joint position data in Cartesian space, and a joint was added from the projection of the center hip joint to the *y* = 0 plane. This phantom joint is very close to the origin (*x* = 0, *y* = 0, *z* = 0), and is generated for training purposes so that the network learns the local body motion but does not learn translational motion. We characterize the skeletal data as four body quadrants, which we use for error analysis in Section VI-C (Fig. 6).

Not all of the datasets we tested on were congruent to the simplified 21 joint CMU structure. The extended MHAD dataset, the formation of which will be discussed in Section IV, is based on a 16 joint model (Fig. 5(b)), and the OSU dataset is based on a 6 joint model (Fig. 5(c)). For testing with these skeletal models, unnecessary joints were omitted from the 21 joint model (Fig. 5(a)) and the network was retrained. Thus, after adding the projection of the center of the hips to the floor, the network was trained three times, once for a 22 joint system, once for a 17 joint system, and once for a 7 joint system.

The network loss was based on a squared *L*^2^ norm (∥·∥_2_) of the difference between the input **X** and the approximation of the input $\hat{X}$. This is done to match the output of the autoencoder as closely as possible to the input thereby learning the most essential aspects of the human motion in the latent space of the network. Also incorporated in the loss function is *L*^1^ (∥·∥_1_) sparsity regularization of the weights and biases, *θ*, with a controlling hyper parameter, *α*. Further regularization was performed in the form of a training dropout rate of 0.2. This training dropout is not to be confused with the data drop-out discussed in the introductory paragraph of Section III. Data drop-out is the method used to corrupt ground truth Mocap data and training dropout is the method used in training in which a certain percentage of neurons are ignored during the update step of training. The Loss,

(17) $$\text{Loss}(X,\theta )=\|X-\Psi (\Phi (X)){\|}_{2}^{2}+\alpha \|\theta {\|}_{1},$$

is optimized with the Adaptive Moment Estimation (Adam) algorithm [28] using moments of 0.9 and 0.999 and a learning rate of 0.001. Equation (17) is written in such a way that the encode and decode operations are explicit so that the operation of the autoencoder is depicted in the equation.

## V. REFERENCED D-MOCAP GENERATION FROM MHAD

**FIGURE 7.**
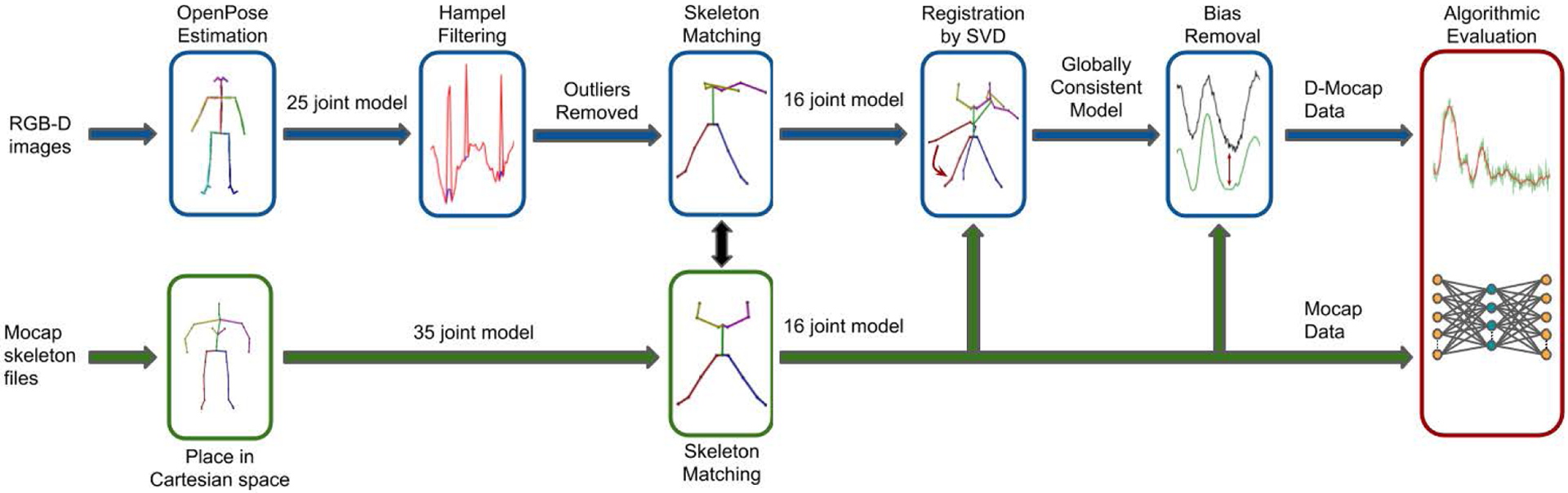
The processing pipeline to repurpose the MHAD dataset (RGB-D and Mocap data) to include D-Mocap data.

Due to the lack of a common dataset, researchers in human motion enhancement have been validating their work in two ways. The first method is the corruption of Mocap data which can be compared to the original data after enhancement [22], [23], [29], [30], [34], [57], [61]. The second method is to capture D-Mocap in the lab along with time-synced Mocap for reference [14], [22], [29], [30], [34], [35], [61]. This method is more indicative of real-world performance but data are often not available to the public and not directly related to existing human motion databases. Thus, we aim to create an extensive dataset that is fully referenced and contains D-Mocap time-synced with optical Mocap. The MHAD dataset is rich in motion data containing RGB-D images captured with two Kinect cameras on 12 human subjects performing 11 actions. The RGB-D images were recorded simultaneously with an Impulse optical Mocap system and time-synced for use as a ground truth comparison. But, in order to use MHAD, the dataset had to be expanded and repurposed for human motion enhancement by generating D-Mocap from these depth images (Fig. 1). The D-Mocap data was generated from the RGB-D images using a five-step process (Fig. 7). This process included generation of D-Mocap data using *ros_openpose_rgbd* [13], removal of outliers with Hampel filtering, skeleton matching with the optical Mocap reference data, registration of the data through singular value decomposition (SVD) [3], and finally bias removal.

**FIGURE 8.**
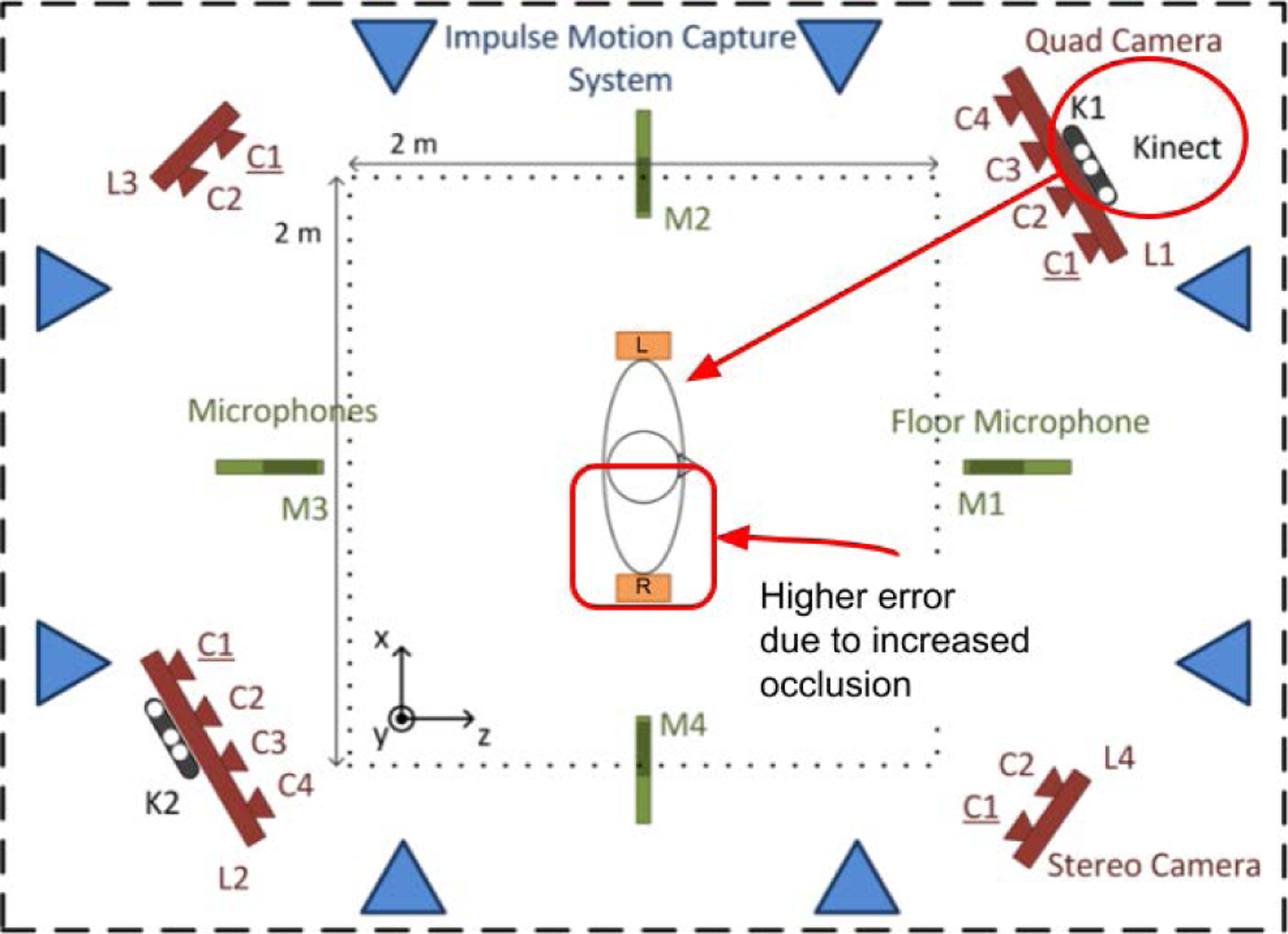
The MHAD dataset is multimodal with off-center Kinect cameras. In generating the MHAD D-Mocap data, this causes a higher amount of occlusion error in the right half of the body. Particularly, in the upper body where motions of higher velocity exist. Image adapted from [42].

The MHAD dataset was chosen due to the fact that it is well respected and used across a wide range of study. MHAD has recently been used in work including human action recognition [21], [45], multi-view and view invariant action recognition [57], human motion synthesis [22], [44], human shape reconstruction [27], and human motion enhancement [23], [30]. In addition, MHAD is multimodal, containing simultaneous recordings by five different systems (Fig. 8).

### A. SKELETON ESTIMATION AND HAMPEL FILTERING

**FIGURE 9.**
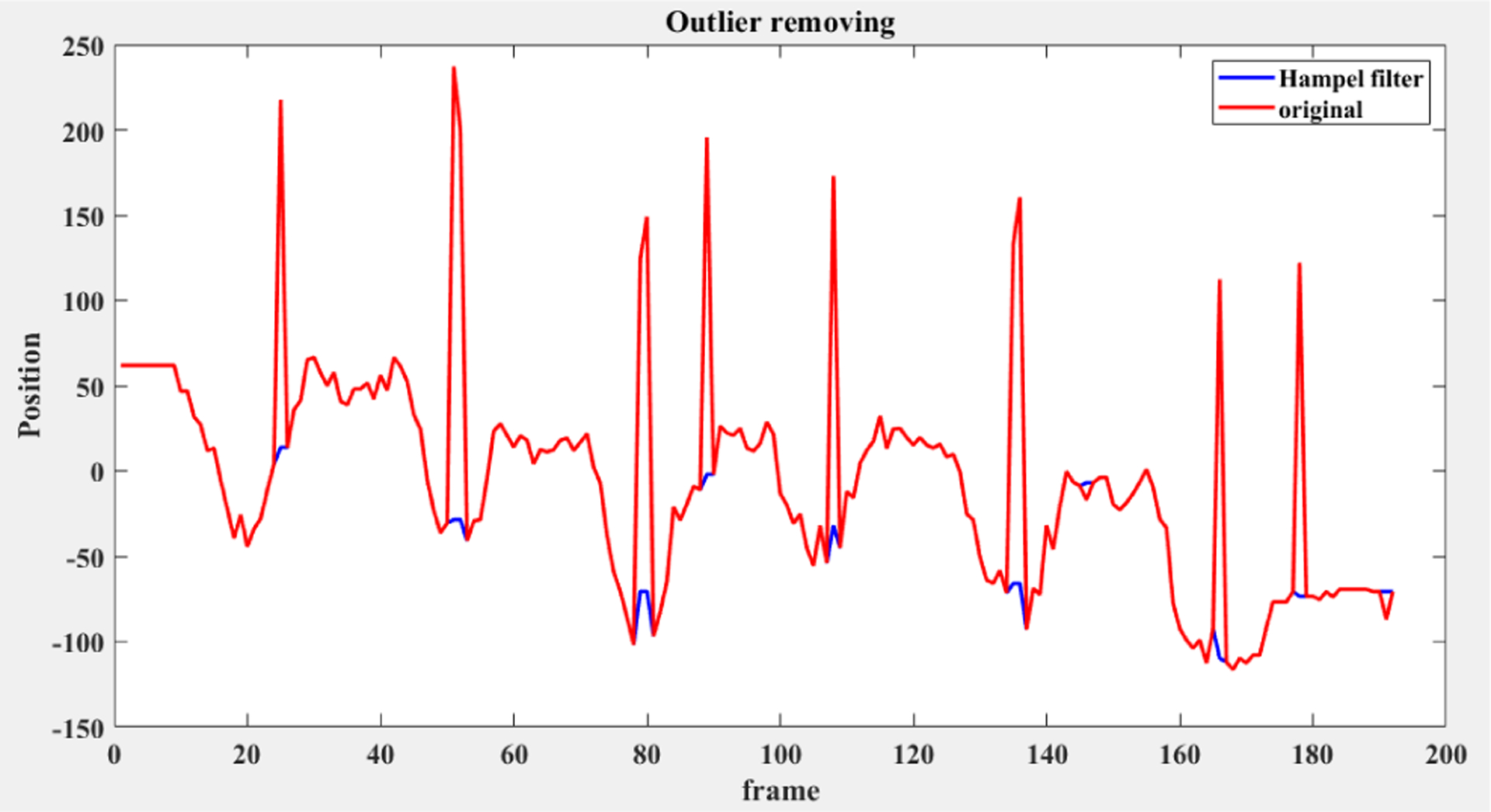
The removal of D-Mocap outliers with Hampel filtering for a 1D motion sequence.

The D-Mocap of our extended MHAD dataset is produced using the RGB-D data in the MHAD dataset and *ros_openpose_rgbd*. *ros_openpose_rgbd* is open source software that extends the well-known Open Pose [11] algorithmto 3-D space using depth information in RGB-D data. Initially, a 2-D skeleton is generated from RGB images with OpenPose and then this skeleton is extended to 3-D space using depth data. This software exhibits the problems we are looking for in D-Mocap data, but also tends to generate extreme outliers in joint position data. These outliers are not typically produced by off-the-shelf SDKs and can skew experimental findings, so they are removed with Hampel filtering (Fig. 9). A Hampel filter is much like a median filter in that a sliding window of seven samples is used and a median is calculated for this window. Standard deviation from the window median can be estimated using median absolute deviation (MAD) where *σ* = 1.4826 MAD. Using this estimation, the joint position datum is replaced by the window median if it is more than three standard deviations away from the median.

### B. SKELETON MATCHING AND REGISTRATION BY SVD

**FIGURE 10.**
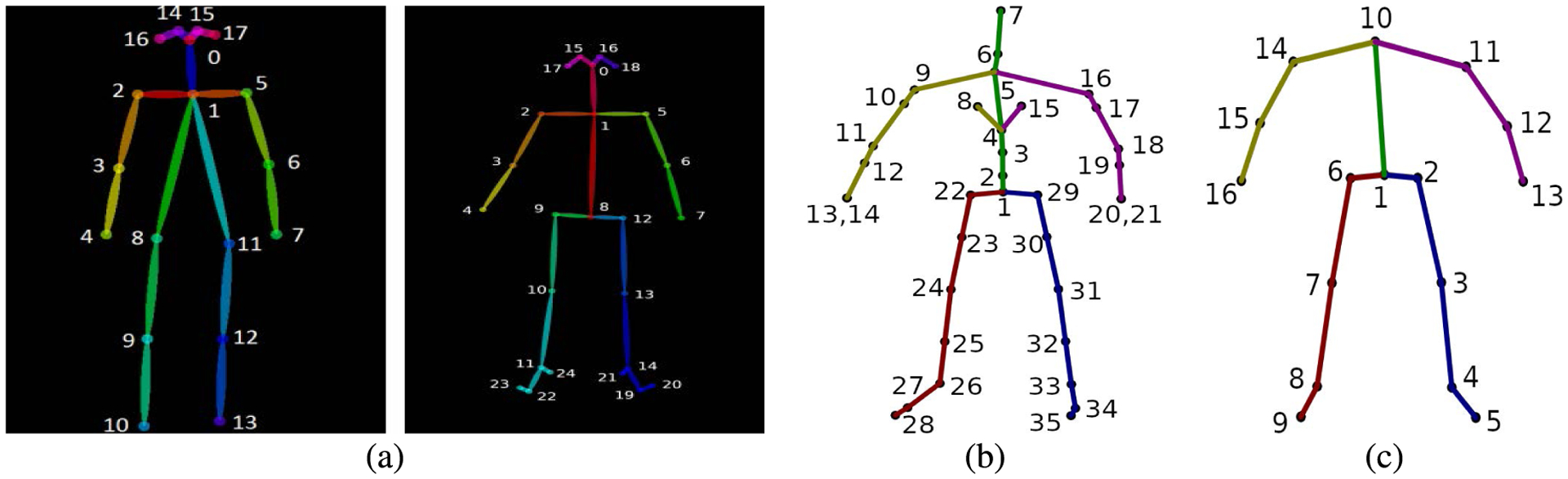
Skeletal model generation for the MHAD D-Mocap data: (a) OpenPose skeletal models (*COCO_18*/*BODY_25*); (b) the skeletal model from MHAD Mocap; (c) the skeletal model after matching *BODY_25* in (a) and (b).

The reference Mocap data provided by MHAD is recorded with a 35 joint skeleton which can be simplified to compare with the *BODY_25* model of OpenPose. The two models share 16 common joints (Fig. 10) which can be directly used in the analysis of human motion enhancement. In contrast, if the *COCO_18* model is used instead of *BODY_25*, there are only 13 joints that can be matched, which omits some representative end effectors like toes that often reflect the effectiveness of filtering and other optimization methods. Though the D-Mocap data and optical Mocap data share the same skeletal model after this skeleton matching, they do not reside in the same Cartesian space and must go through registration. In registration with SVD, a centroid for each set of data is determined using all joint positions for the entire sequence,

(18) $$C=\frac{1}{N\cdot M}\sum_{k=1}^{M}\sum_{j=1}^{N}p_{k,j},$$

where *N* is the number of joints in the skeletal model and *M* is the number of frames. Using (18), an intermediate matrix **A** is calculated from the D-Mocap data $\bar{X}$, Mocap data **X**, and the centroids of each dataset $\left(C_{\bar{X}},C_{X}\right)$,

(19) $$A=(\bar{X}-C_{\bar{X}}){\left(X-C_{X}\right)}^{T}.$$

We can then calculate a rotation matrix **R** and translation vector **t** for the D-Mocap data using the SVD of the intermediate matrix *A* defined in (19):

(20) [U,S,V]=SVD(A),

(21) $$R=UV^{T},$$

(22) $$t=C_{X}-RC_{\bar{X}},$$

where **U**, **S**, and **V** are the unitary and diagonal matrices obtained via SVD in (20). A detailed explanation for the derivation of (19), (21), and (22) can be found in [3]. A rigid body transform can then be performed on the D-Mocap data so that the skeletons are matched as closely as possible in the same 3-D space,

(23) $$\tilde{X}=R\bar{X}+t,$$

where $\tilde{X}$ is the D-Mocap data after registration. The operation in (23) aligns the skeletons by rotating and translating the D-Mocap data without modifying the relative positions of the joints.

### C. BIAS REMOVAL AND NETWORK PREPROCESSING

Finally, since the skeleton models are defined differently, the offset for each joint must be removed so that the D-Mocap can be compared to the optical Mocap. These constant offsets are not a result of occlusion or noise and are a result of how these two skeletal models define their joint positions. Thus this bias can be removed by calculating the difference of each Cartesian coordinate for each joint *j* in each frame *k*, between the D-Mocap data and Mocap reference data. These values are then averaged over all frames using:

(24) $$Bias_{j}=\frac{1}{M}\sum_{k=1}^{M}{\tilde{X}}_{k,j}-X_{k,j},$$

where *M* is the total number of frames, $\tilde{X}$ is the D-Mocap data in Cartesian space, and **X** is the Mocap data in Cartesian space. The calculation in (24) is done for each joint so that the constant offset can be removed and we can more accurately characterize the error between the D-Mocap data and the reference Mocap data.

**FIGURE 11.**
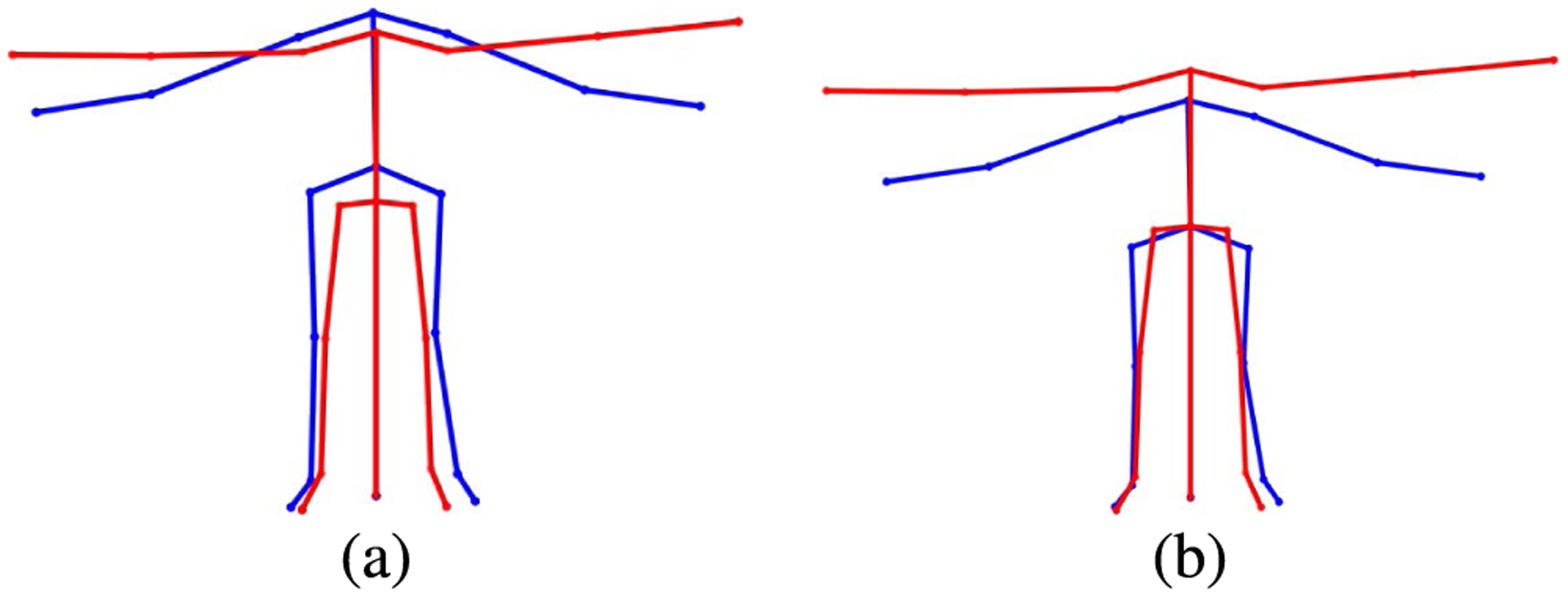
Scaling the MHAD subject skeleton as a preliminary procedure to match the skeleton to the autoencoder training data. The blue and red skeletons are the T-Pose from the training data and an MHAD subject, respectively, (a) before scaling and (b) after scaling.

As discussed previously, the convolutional autoencoder is trained on a homogeneous set of motion clips which have all been retargeted to the same skeletal structure. As a result, the network needs to work with data of this same structure, and will produce results of this skeletal structure as well. Therefore, the MHAD D-Mocap data must be adjusted before passing through the neural network, and must be adjusted once again at the output in order to compare our results with the ground truth Mocap data which adheres to the original skeletal structure. The D-Mocap data comes from a variety of subjects with varying heights and bone lengths. To mitigate the height discrepancy, the data was first scaled by calculating the mean distance from the center of the hips to the floor, *y* = 0 plane, over all of the frames of that subject’s T-Pose clip (Fig. 11). The ratio between this distance and the hip-to-floor distance of the T-pose of the simplified CMU 21 joint skeleton (Fig. 5(a)), is used to scale the subject’s skeleton.

Once scaled, the test data are matched to the skeletal structure of the training data by optimizing the bone lengths of the D-Mocap data to conform to the known bone lengths of the training data. Since the training data are homogeneous, these lengths are known and are constant. Thus the D-Mocap data are optimized over the latent space of the convolutional autoencoder with a bone constraint before recovery, which ensures the D-Mocap skeleton adheres to the skeletal structure of the training data. Given that the Cartesian joint positions at the endpoints of bone *b* at frame *k* are represented by **b**_*m*,*k*_ and **b**_*n*,*k*_, the constraint that is optimized is given by

(25) $$\text{Bone}(\tilde{X})=\sum_{k}\sum_{b}{\left|{\left\|b_{m,k}^{H}-b_{n,k}^{H}\right\|}_{2}-l_{b}\right|}^{2},$$

where $b_{m,k}^{H}$ and $b_{n,k}^{H}$ are the mappings of the joints at the two ends of bone *b* into the latent space at frame *k* as done in (16), and *l*_*b*_ is the known bone length of bone *b* from the training data. After matching the skeletal structure to that of the training data using (25), a phantom joint is added at the projection of the center of the hips on the *y* = 0 plane. Thus the MHAD skeleton becomes a 17 joint model for use with the autoencoder. After recovery, the D-Mocap data are inversely scaled to their original size and the bias between the skeletal structures is removed using (24). This methodology was chosen over retargeting in order to keep the reference Mocap data unmodified, as retargeting to the skeletal structure of the training data would require the retargeting of the reference Mocap as well as the D-Mocap.

As an initial cursory investigation of the extended MHAD dataset, we examined the results of four recent methods of human motion enhancement, including three nonlinear Kalman filtering (KF) methods [61], namely the EKF, the UKF, the TKF, and a convolutional auto encoder [23]. These results prove a good baseline for our new extended MHAD dataset and are reported on a joint-by-joint basis in Table 1.

## VI. EXPERIMENTAL RESULTS

To ensure robust analysis, our two proposed algorithms (TKF-assisted and TKF-refined autoencoders) were evaluated on two types of test data, simulated and real-world D-Mocap data. In the following, we will first discuss three datasets used for testing, then the processing of D-Mocap data captured by the two labs at OSU, and the experimental results of using these datasets.^2^

### A. TESTING DATA OVERVIEW

Simulated data were derived by corrupting the CMU Mocap data with two types of noise at two different levels. First, CMU data were corrupted with zero mean additive Gaussian white noise (AWGN) at standard deviations of 7 cm and 10 cm. Secondly, data were randomly removed from the CMU data at two different rates, 25% and 50%. This data drop-out was quite excessive and was better handled by the autoencoder than the TKF. Two data sets of real-world D-Mocap data were used for robustness in analysis. The first was captured at OSU by the Biomechanical Analysis and Musculoskeletal Modeling (BAMM) Lab and the Visual Computing and Image Processing Lab (VCIPL). The second set of D-Mocap data was the extended MHAD dataset discussed in the previous section. These testing sets consist of three different skeletal structures (Fig. 5) and prove the flexibility of our method for varying skeletal models.

The convolutional autoencoder central to our work is trained on a retargeted homogeneous skeleton which presents a challenge if it is to be used on motion data that is not of matching skeletal structure. The simulated data derived from corrupting the CMU dataset is of the same structure as the training data before corruption, so no additional processing is necessary for this analysis. However, the other two real world datasets must undergo some processing in order to be analyzed with regard to their respective Mocap ground truth references. The OSU real-world dataset is discussed briefly here as was done in the previous section for MHAD since it differs from the structure of the retargeted CMU skeleton.

### B. OSU D-MOCAP DATA PROCESSING

The OSU D-Mocap dataset was originally created for gait assessment and therefore concentrates on the six most important joints at the lower-body. These joints being the hip, knee, and ankle joints [4], [39]. The depth data for this dataset were captured with an Orbbec Astra and processed with the Nuitrack SDK to produce the OSU D-Mocap. An Optitrack Mocap system was used to capture marker data side-by-side to the D-Mocap data. The motions captured in this dataset were all gaits from the same subject that consisted of three distinct walking motions.

**FIGURE 12.**
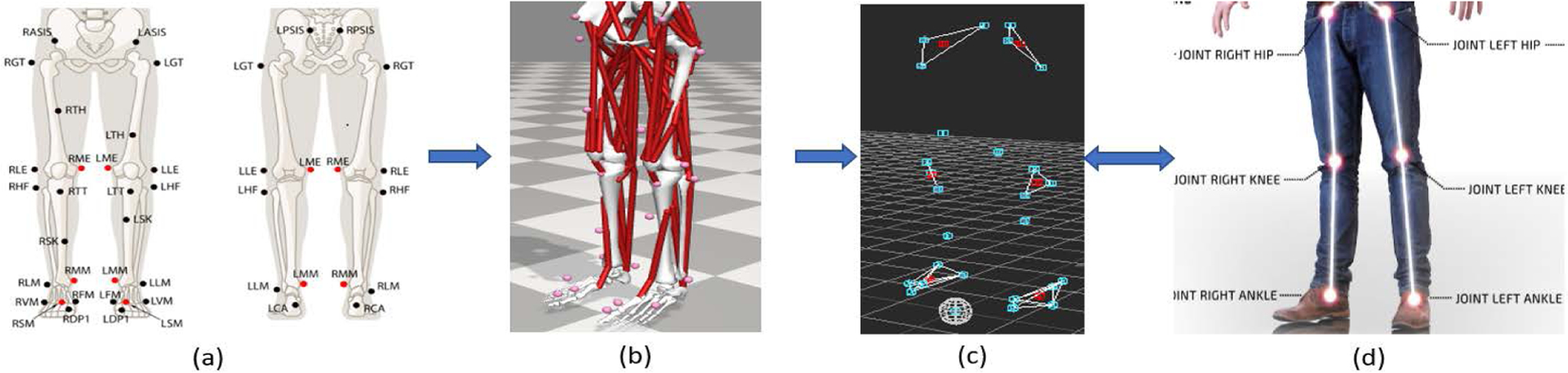
The process for creating internal joint data from Mocap markers for the OSU dataset: (a) OptiTrack marker definitions; (b) OptiTrack marker positions; (c) MotionBuilder marker positions with centroids; (d) Nuitrack joint positions.

The Mocap collected from the Optitrack system was in the form of 32 external marker positions (Fig. 12(a)) and (Fig. 12(b)), and the D-Mocap data collected was in the form of internal joint positions (Fig. 12(d)); therefore, they could not be directly related. We used MotionBuilder software to restore any missing marker data and created rigid bodies with the markers nearest to joints of interest. We then calculated the centroids for these rigid bodies to be used as the internal joints of the Mocap data (Fig. 12(c)).

In order to accommodate the OSU data that only cover six lower-body joints, the convolutional autoencoder was retrained with the retargeted CMU data by omitting all joint data other than joints 2, 3, 4, 6, 7 and 8 from (Fig. 5(a)). The D-Mocap data captured from the Nuitrack SDK was then size matched to the training data using the distance from the hips to the floor of both skeletons. The autoencoder is biased toward generating recovery motion of the same skeletal structure as the training data. Thus, before the resultant OSU data can be effectively compared to the Mocap reference ground truth, this bias must be removed. This bias is not related to error in motion estimation but rather just a difference in the skeletal structure of the OSU subject and the CMU subject. After the data are recovered from the autoencoder it is first re-scaled using the inverse ratio of hip-to-floor measurements that it was originally scaled with. Next, bias removal is done using (24) as was done with the MHAD data.

### C. QUANTITATIVE EVALUATION

**FIGURE 13.**
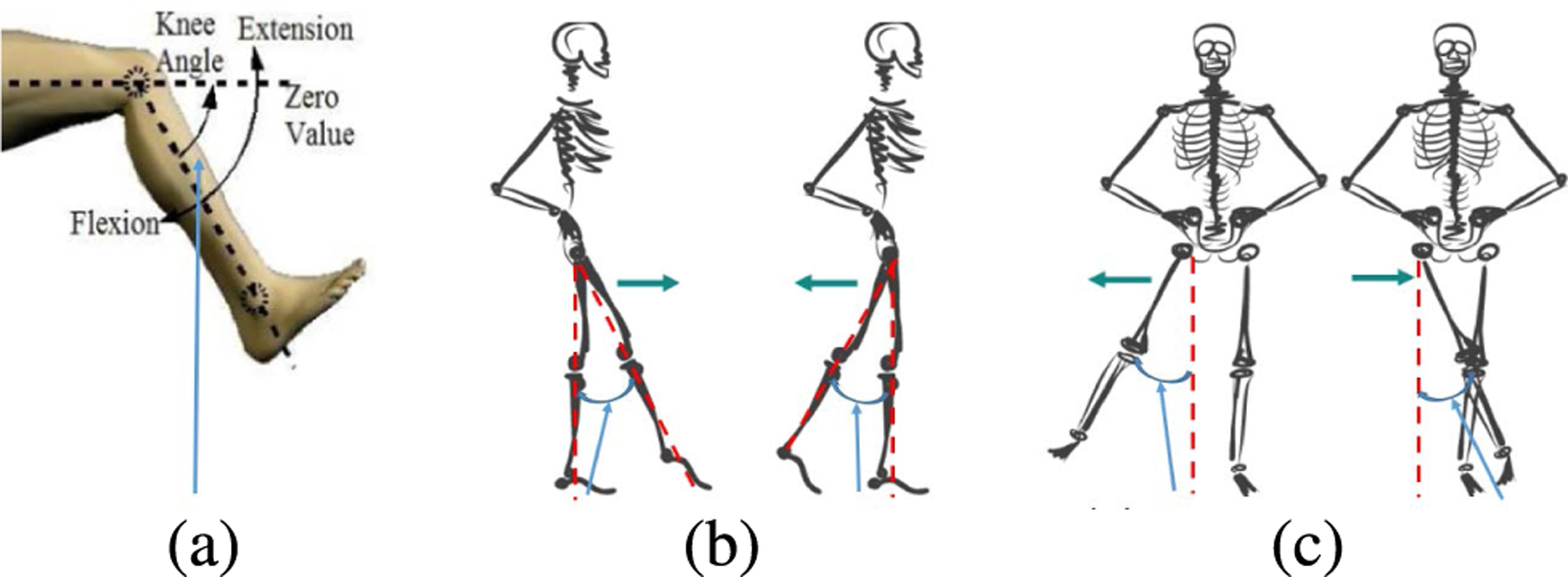
Joint angle definitions used in gait analysis: (a) Knee flexion angle (LKF/RKF); (b) hip flexion angle (left, LHF) and Hip extension angle (right, RHF); (c) hip abduction angle (left, LHA) and hip adduction angle (right, RHA).

We use three metrics to quantify the results of our work. The first metric is the Euclidean distance of each joint position from its respective ground truth joint position. These distances are averaged over all frames of motion to yield a joint-by-joint average distance from ground truth (RMSE). In order to succinctly analyze our results, these joint-by-joint values are averaged over all joints in order to arrive at a single mean Euclidean distance for the skeleton as a whole. The second metric is the bone length error using the corresponding bone length from the reference Mocap data:

(26) $$Err^{\left(b\right)}=\frac{1}{M}\sum_{k=1}^{M}\left|{\left\|b_{m,k}^{\left(b\right)}-b_{n,k}^{\left(b\right)}\right\|}_{2}-{\left\|l_{m,k}^{\left(b\right)}-l_{n,k}^{\left(b\right)}\right\|}_{2}\right|,$$

where *Err*^(*b*)^ is the error of the length of bone *b* averaged over *M* frames, $b_{m,k}^{\left(b\right)}$ and $b_{n,k}^{\left(b\right)}$ the estimated joint positions, and $l_{m,k}^{\left(b\right)}$ and $l_{n,k}^{\left(b\right)}$ the Mocap reference ones at the two ends of bone *b*. In this way, (26) is used to quantify an average diversion from the known bone lengths of the reference Mocap data. In addition, human motion is analyzed with regard to five lower body joint angles that are often used for gait assessment [4], [39], an important field that could greatly benefit from the use of depth sensors rather than optical Mocap systems. The angles studied for both the right and left legs are knee flexion angle (LKF/RKF), hip flexion angle (LHF/RHF), hip extension angle (LHE/RHE), hip abduction angle (LHAB/RHAB), and hip adduction angle (LHAD/RHAD). The definitions of these joint angles are represented in Fig. 13. In the interest of simplicity, the hip flexion and extension angles have been combined (LHF/RHF) as have the hip abduction and adduction angles (LHA/RHA).

We provide results on these metrics for the following: unaffected low-quality Mocap data, data enhanced with the autoencoder only, data enhanced with the TKF only, data enhanced using KF-assisted autoencoder, and data enhanced using TKF-assisted autoencoder (real-world D-Mocap and simulated D-Mocap with AWGN corruption) and TKF-refined autoencoder (simulated D-Mocap with data drop-out corruption). Over 100, 000 frames of corrupted data were tested in each of the simulation data groups. The size of the real-world D-Mocap datasets used were 147, 390, and 2, 800 frames for the extended MHAD dataset and OSU dataset respectively.

#### 1) JOINT POSITION ERROR

Table 2 serves as a synopsis of our results with regard to joint position error and provides joint position RMSE averaged over all joints. In the interest of space, for the following tables we will refer to the KF-assisted autoencoder, TKF-assisted autoencoder, and TKF-refined autoencoder methodologies as KF-A, TKF-A, and TKF-R respectively. Here we see the autoencoder performs well on simulation data as the motion manifold easily removes the noise that is very uncharacteristic to human motion. The TKF-assisted/TKF-refined methods improve the results of the autoencoder and the TKF, which shows that our methodology is a copacetic merger of the kinematic information contained in the TKF and the human motion manifold contained in the neural network. The autoencoder does not perform as well on the real-world D-Mocap data as it does not have a noise component that is as easily extracted as the simulation noise. Here the TKF-assisted method shows improvement over both the autoencoder and the TKF and goes to show that the autoencoder benefits from the preservation of the kinematic aspects of the original data.

#### 2) BONE LENGTH ERROR

Table 3 provides a synopsis of average bone length error over all testing frames. It is worth noting the benefits of using the autoencoder which is based on a homogeneous skeleton with constant bone length. The neural network is trained on a consistent structure so the results of the autoencoder are also consistent and benefit from the fact that the learned human motion manifold adheres to a constant bone length. Interestingly, when a KF-assisted autoencoder is used, the bone length results are hindered as the KF assumes a linear system and it is not working synergistically with the autoencoder. However, the TKF is more amenable to the structure of the learned motion manifold, resulting in a more consistent bone structure. As a whole, these three tables show that our two algorithms (TKF-assisted/TKF-refined) consistently improve on both the results of the autoencoder and the TKF when they are used alone. It is also shown that the TKF is more kinematically accurate and amenable to the motion manifold compared with the KF.

#### 3) JOINT ANGLE ERROR

Table 4 provides joint angle errors averaged over all testing frames and averaged over all 6 joint angles. In addition, we provide an angle-by-angle joint angle error analysis on all six joint angles discussed previously in this section. These individual joint angles are important to the field of gait assessment which could benefit from a human motion capture method using RGB-D data instead of optical motion capture systems. Because of this interest in depth sensors as a newly viable method for gait assessment we have chosen to include a breakdown of our findings rather than averages as shown in Table 4. Table 5 provides the average joint angle error for each angle using the three test datasets. We see the TKF is able to work harmoniously with the hours of quality training data from the autoencoder, as well as preserve the kinematics of the motion data resulting in joint angle errors (around 3° ~ 5°) near those achieved by inertial sensors [8]. This is important as the field of gait analysis could benefit from the freedom of using a markerless system that is not tied down to a particular area and is not dependent on the restrictive markers that other systems require.

#### 4) BODY QUADRANT ANALYSIS USING THE EXTENDED MHAD DATASET

We chose to adaptively adjust the thresholds of the TKF using joint velocity for two reasons. First, actions of higher velocity increase the possibility of estimation error since the joint positions are rapidly changing from frame to frame. Secondly, occlusion error is often accompanied by a rapid shift in velocity as the joint re-emerges. Fortunately the MHAD dataset provides us with a unique tool to analyze the benefits of using the TKF-assisted autoencoder to alleviate these problems. This is because the majority of motions in the MHAD dataset are upper body motions, and the front-facing Kinect camera is placed at an angle with reference to the human subject, and thereby obscures the right half of the body slightly. This results in a greater frequency of occlusion errors in joint estimation (Fig. 8). Using this knowledge, we can confirm actions of higher velocity increase the possibility of estimation error, the off-angle camera placement causes occlusion which increases joint position error and joint velocity, and that adaptively adjusting the limits of the TKF using joint velocity is critical in mitigating these problems.

##### a: ACTIONS OF HIGHER VELOCITY INCREASE THE POSSIBILITY OF ESTIMATION ERROR

Table 6 depicts the results of enhancing D-Mocap from the extended MHAD dataset with respect to four quadrants of the body (Fig. 6). This dataset is taken from approximately 82 minutes of RGB-D data from the front-facing Kinect camera in the MHAD dataset. We see in Table 6, that when the upper body is isolated, the average RMSE is 3.9 cm worse than that of the lower body and the average joint velocity of the MHAD reference is 4.8 cm/s greater in the upper body. The upper body has a higher average velocity component due to most of the subject’s movement being in the upper body and this increases the possibility of estimation error.

##### b: OCCLUSION INCREASES JOINT POSITION ERROR AND VELOCITY

Because of the off-angle placement of the Kinect camera in the MHAD dataset, we expect to see a higher joint RMSE on the right side of the body due to occlusion. When considering the upper body which contains the most significant movement, we see the average RMSE of the upper right quadrant of the body is 1.3 cm worse than that of the upper left quadrant. Moreover, the mean joint velocity in the upper right quadrant increases 82.5% when compared to Mocap data and the upper left only increases 70% (Table 6). The reason for the larger increase in the right half of the body is largely due to the occlusion error caused by the off-angle position of the Kinect camera, which slightly obscures the right portion of the body. Thus, we see that occlusion errors increase the velocity component of joints in D-Mocap data.

##### c: ADAPTIVE CONTROL OF THE TKF USING JOINT VELOCITY MITIGATES THESE ISSUES

We have adaptively adjusted the limits of the TKF using joint velocity to remedy estimation error since joint occlusion is often accompanied by large shifts of velocity, and joints with a higher velocity component are more prone to estimation error. Therefore, we should see the largest improvement in our TKF-assisted autoencoder in the section of the body that has the largest content of joint position error. This section of the body should also have the largest increase of joint velocity associated with it. We find that this is indeed the case as we see the greatest improvement in RMSE, 37.2 cm, in the upper right quadrant of the skeleton, which has the highest joint position RMSE, 11.7 cm, and the highest increase in joint velocity, 37.8 cm/s.

### D. QUALITATIVE EVALUATION

**FIGURE 14.**
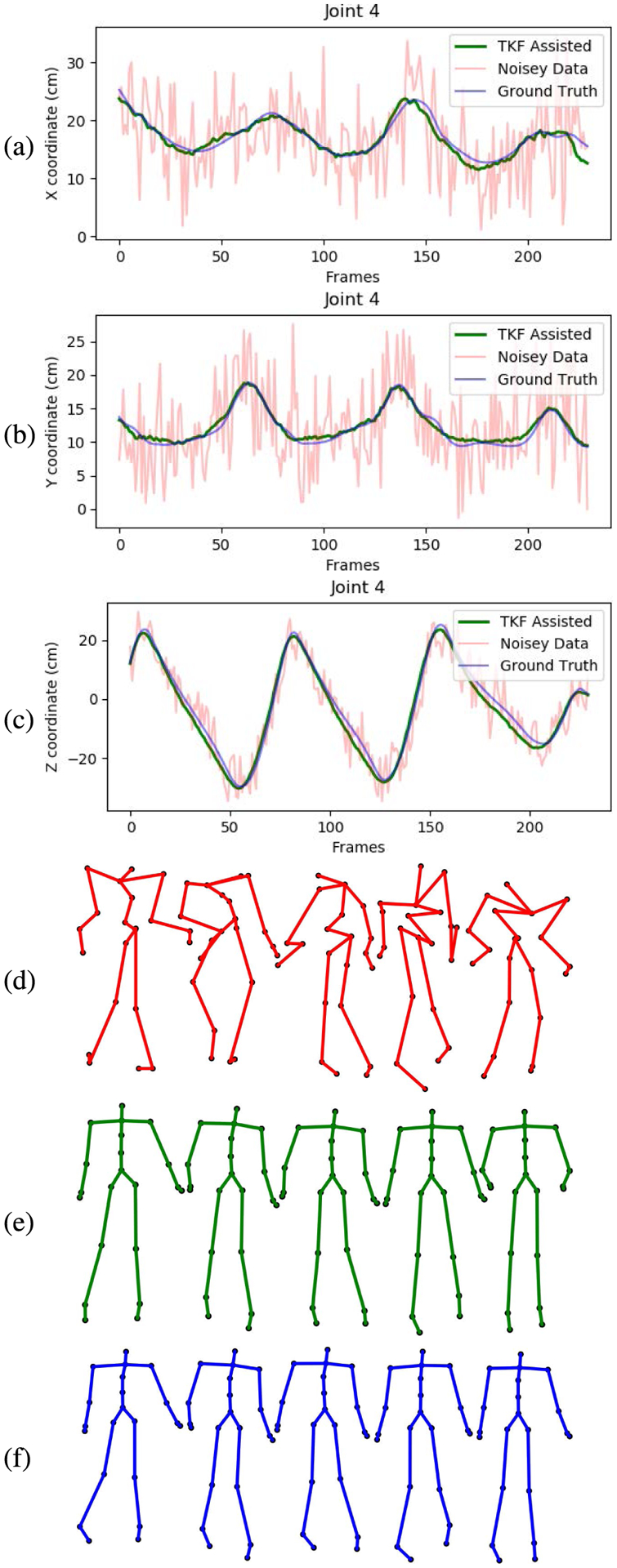
The Cartesian coordinates of the left ankle of CMU dataset, subject 8, trial 4. Depicted are (a) X coordinate, (b) Y coordinate, and (c) Z coordinate. Original Mocap data are depicted in blue, Mocap data corrupted by AWGN in red, and the TKF-assisted recovery in green. Corresponding frames of motion are depicted in (d) data corrupted by AGWN, (e) TKF-assisted recovery, and (f) Mocap reference data.

**FIGURE 15.**
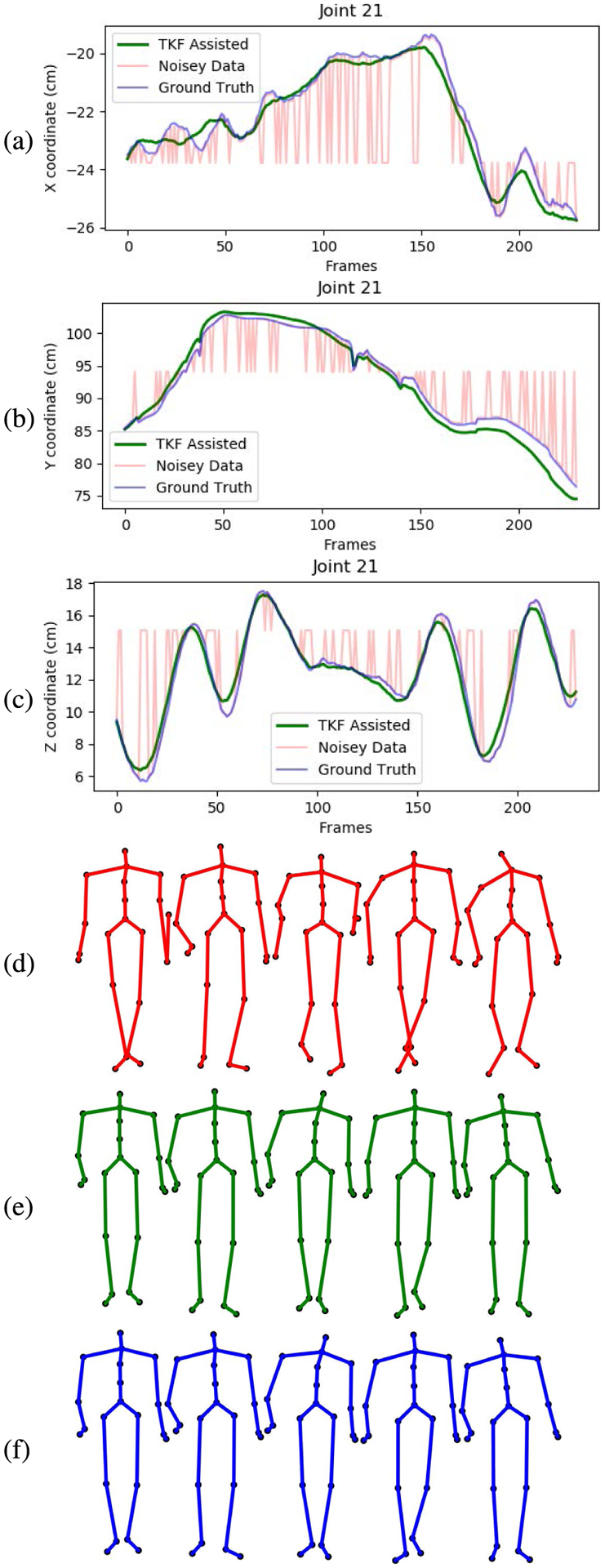
The Cartesian coordinates of the right hand of CMU dataset, subject 115, trial 10. Depicted are (a) X coordinate, (b) Y coordinate, and (c) Z coordinate. Original Mocap data are depicted in blue, Mocap data corrupted by AWGN in red, and the TKF-assisted recovery in green. Corresponding frames of motion are depicted in (d) data corrupted by data drop-out, (e) TKF-assisted recovery, and (f) Mocap reference data.

**FIGURE 16.**
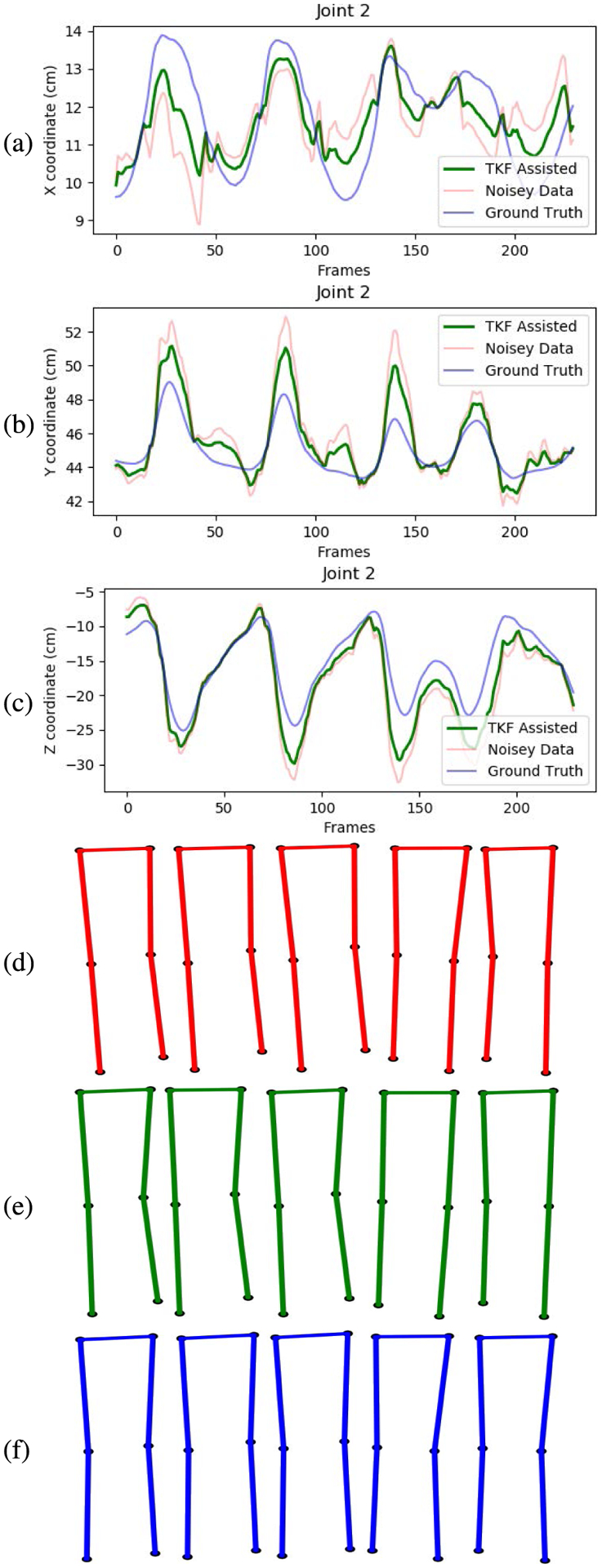
The Cartesian coordinates of the left knee of OSU dataset normal walk motion. Depicted are (a) X coordinate, (b) Y coordinate, and (c) Z coordinate. Original Mocap data are depicted in blue, Mocap data corrupted by AWGN in red, and the TKF-assisted recovery in green. Corresponding frames of motion are depicted in (d) D-Mocap, (e) TKF-assisted recovery, and (f) Mocap reference data.

**FIGURE 17.**
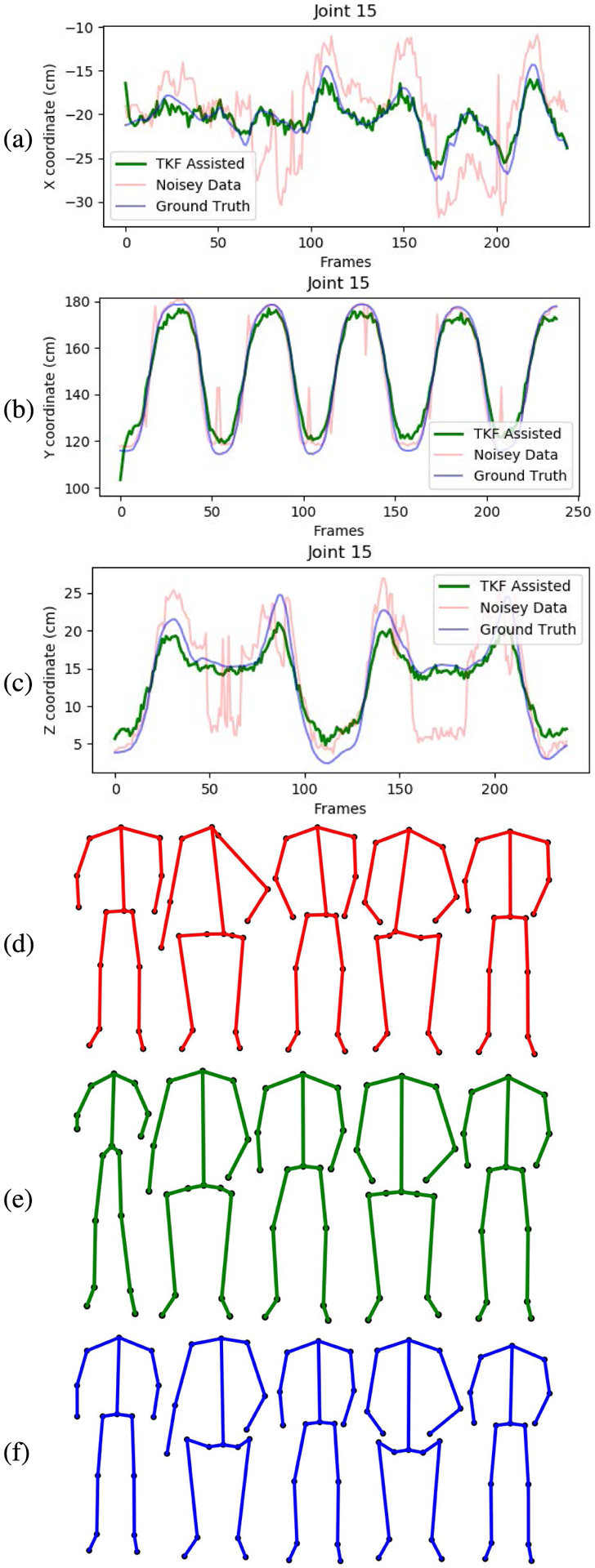
The Cartesian coordinates of the right elbow of MHAD dataset, subject 3, action 9, repetition 1. Depicted are (a) X coordinate, (b) Y coordinate, and (c) Z coordinate. Original Mocap data are depicted in blue, Mocap data corrupted by AWGN in red, and the TKF-assisted recovery in green. Corresponding frames of motion are depicted in (d) D-Mocap, (e) TKF-assisted recovery, and (f) Mocap reference data.

**FIGURE 18.**
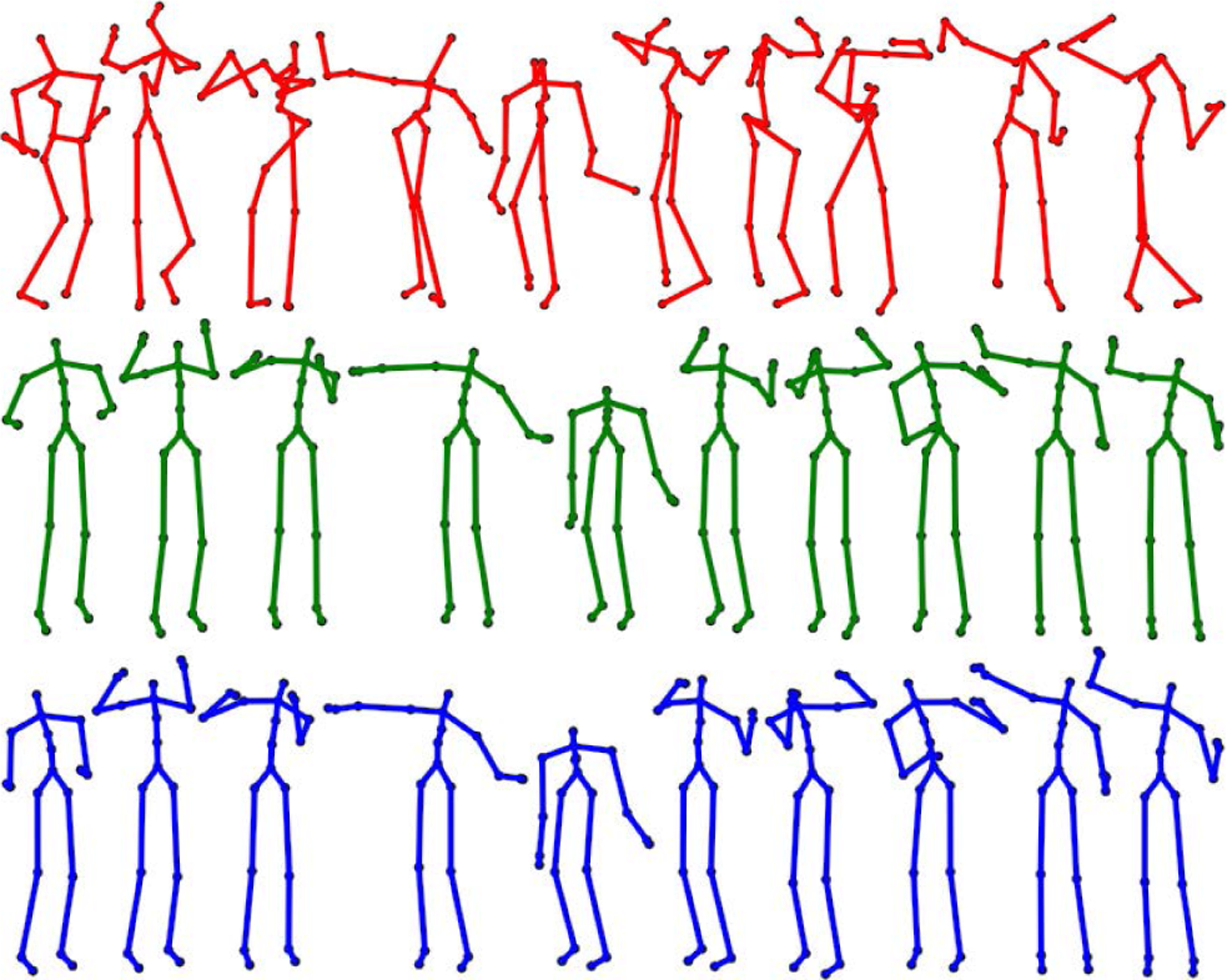
Motion from subject 12 trial 4 of the CMU Motion Capture Database. Mocap reference is depicted in blue, Mocap affected with AWGN is depicted in red, and the TKF-assisted recovery is depicted in green.

**FIGURE 19.**
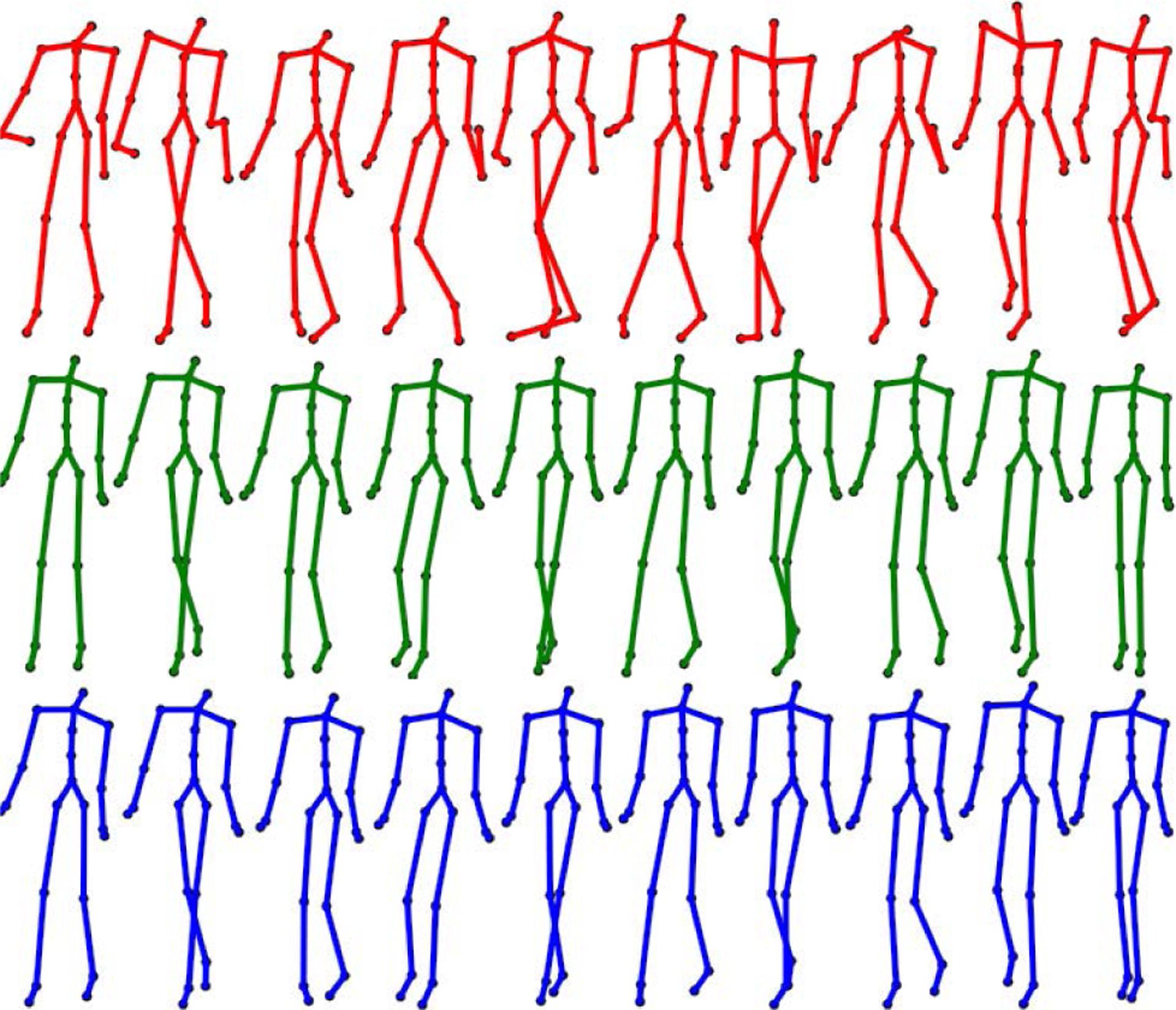
Motion from subject 8 trial 1 of the CMU Motion Capture Database. Mocap reference is depicted in blue, Mocap affected with data drop-out is depicted in red, and the TKF-assisted recovery is depicted in green.

**FIGURE 20.**
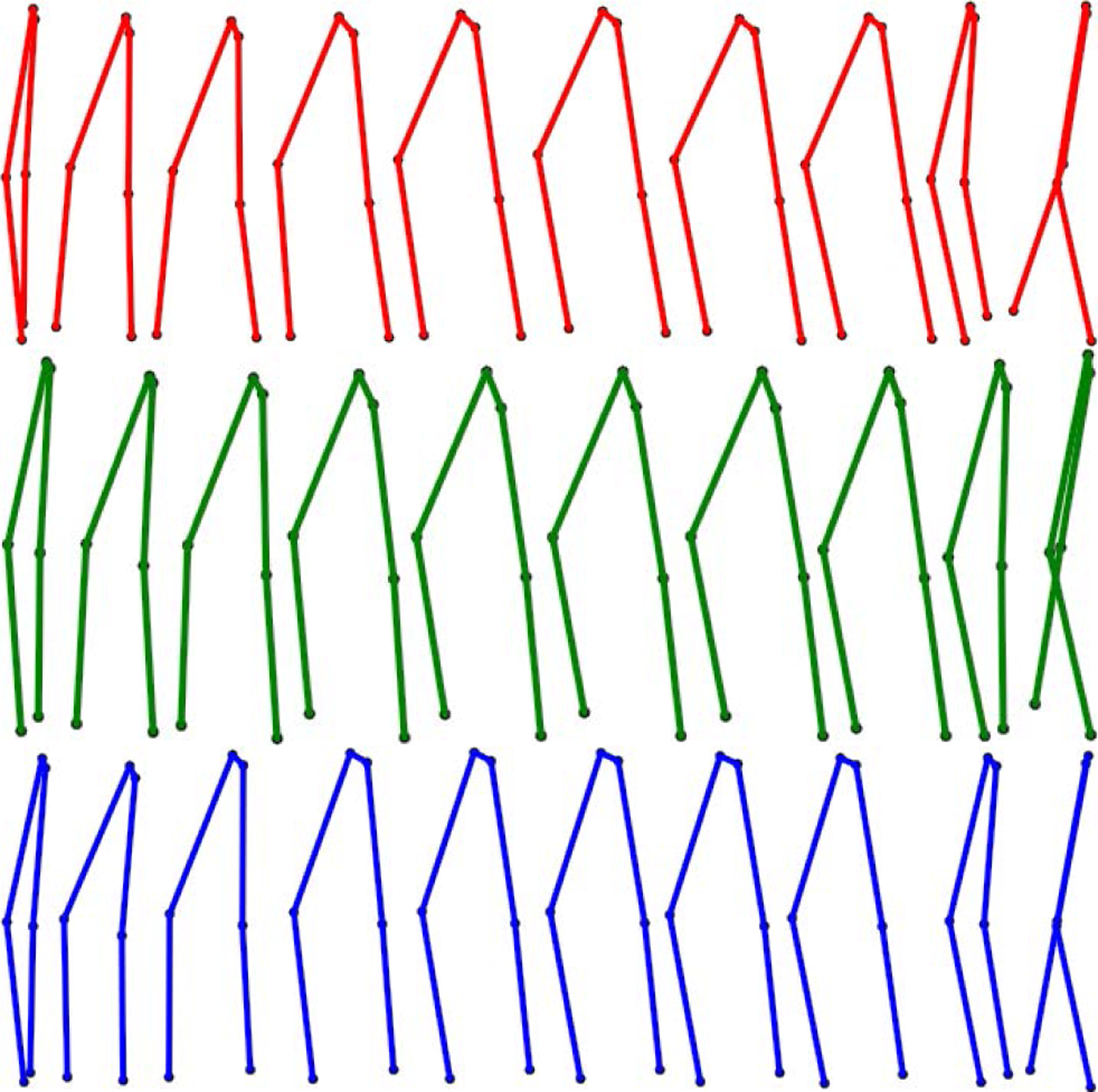
Motion from the elongated step motion of the OSU dataset. Mocap reference is depicted in blue, D-Mocap is depicted in red, and the TKF-assisted recovery is depicted in green.

**FIGURE 21.**
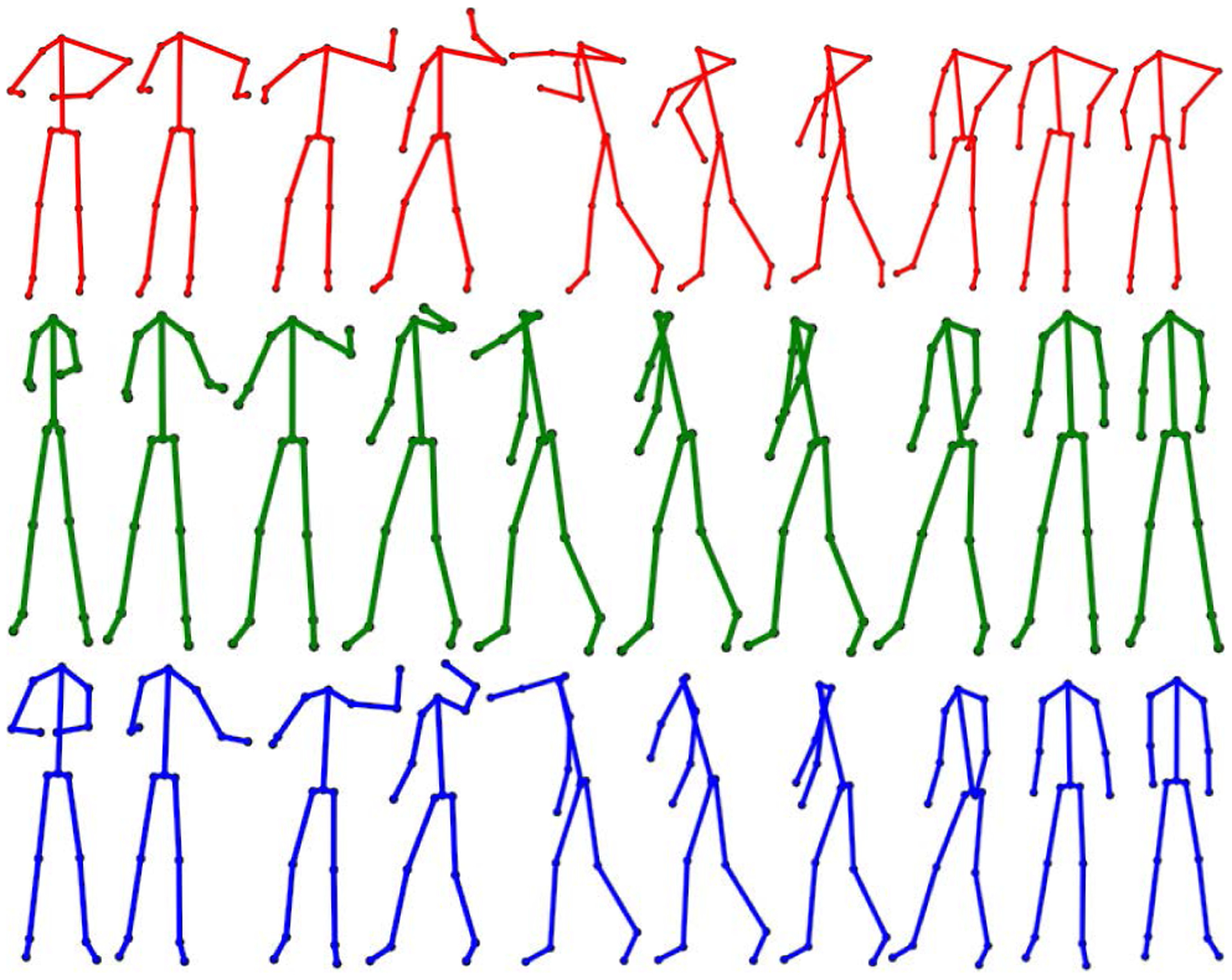
Motion from subject 9, action 8, repetition 1, of the extended MHAD Database. Mocap reference is depicted in blue, D-Mocap is depicted in red, and the TKF-assisted recovery is depicted in green.

The smoothing and recovery effects of the TKF-assisted/TKF-refined autoencoder algorithms on the lower quality Mocap data are depicted in (Figs. 14, 15, 16, and 17). The Cartesian coordinates of the left ankle corrupted by AWGN are shown in (Fig. 14). Likewise, the results on the right hand of simulated data drop-out, those of the left knee in the OSU D-Mocap data, and those of the right elbow in the MHAD data are shown in (Figs. 15, 16, and 17) respectively. In addition, longer frame sequences are shown in (Figs. 18, 19, 20, and 21). From these figures we can see that the proposed two algorithms successfully enhance the lower quality D-Mocap data and make them closer to the corresponding ground truth joint positions. The images associated with the simulation data (corrupted Mocap data) are quite striking because the autoencoder is very efficient at reducing noise and outliers that are common in real-world D-Mocap data.

### E. RUN-TIME EVALUATION

The TKF-assisted method was evaluated on two systems working in parallel. One system to create target TKF data, and one system to optimize the recovered motion in the latent space of the autoencoder. The first system used an Intel Core i7-4770 CPU with 16GB of memory, and all the TKF code was written in MATLAB. The second system used an Intel Core i7-8700K CPU with 32GB of memory and a GeForce GTX 1080 GPU with 8GB of memory. The autoencoder and all optimization code was written in Python 3.9 with the Theano 1.0.5 library [52]. Due to the nature of our method and the need for a complete motion target for optimization in the latent space, real-time operation is not possible. Table 7 compares the run-time of several recent human motion enhancement methods. The table is split into real-time methods and off-line methods and the hardware and software used in evaluation is included where reported in literature. Run-time values are given in seconds per frame and are either reported directly or calculated from literature using the length of the testing data and the reported run-time.

## VII. CONCLUSION

We present a novel approach to human motion enhancement for low-quality D-Mocap data, and our goal is to make low-cost depth sensors a practical and viable motion analysis tool for clinical applications where the optical Mocap systems may not be plausible. The advantage in our approach is the synergistic melding of the two complementary elements, TKF-based filtering and autoencoder-based manifold learning. The former is able to preserve the kinematics properties of joint trajectories by involving a unique Tobit model to handle censored measurements mainly caused by occlusion or interference. The latter is used to take advantage of the rich and diverse high-quality CMU Mocap data by learning a general motion manifold to capture the joint spatial-temporal structure of skeleton-based motion data. To accommodate different types of data corruption, two paradigms, the TKF-assisted and TKF-refined autoencoders, have been proposed. The first involves latent space optimization of two elements that is able to handle noisy motion, and the second one is a serial connection of the two elements that is suitable for missing data handling with data drop-out. We have created an open source D-Mocap dataset by extending the MHAD database and including our self-collected OSU data that is intended to promote human motion enhancement research in the community. Our proposed algorithms have been evaluated and compared on both real-world and simulated D-Mocap datasets, and experimental results demonstrate that motion data have been significantly improved in terms of both kinematics (joint positions/angles) and anthropometrics (bone lengths). Our future research will focus on the applicability and relevance of improved D-Mocap for clinical biomechanics study.

## Figures and Tables

**TABLE 1.** Baseline results for four human motion enhancement methods using the extended MHAD dataset (cm).

Joint Indices	D-Mocap Data	EKF Method [61]	UKF Method [61]	TKF Method [61]	Autoencoder Method [23]
1	1.5	3.2	2.4	2.0	2.2
2	3.6	4.1	2.6	2.9	2.4
3	3.4	4.6	3.1	2.9	4.0
4	3.7	4.2	3.7	3.0	4.7
5	6.7	5.6	4.0	4.2	7.5
6	3.6	4.5	2.6	2.9	2.4
7	4.0	4.1	3.3	3.0	4.4
8	4.2	4.7	3.9	3.2	5.0
9	8.7	5.6	4.0	5.3	7.5
10	5.2	5.6	6.1	4.3	4.0
11	5.2	6.4	7.9	4.4	4.2
12	8.2	7.8	8.0	6.7	6.2
13	19.4	13.6	13.3	11.6	13.2
14	10.7	6.2	7.0	6.3	6.0
15	16.1	11.0	10.9	9.9	11.7
16	30.4	17.0	17.0	16.3	19.5
Ave.	8.4	6.7	6.2	5.5	6.6

**TABLE 2.** Average joint RMSE for three datasets (cm).

Data Type	Simulation					
	Corrupted Data	Autoencoder Only [23]	TKF Only [61]	KF-A	TKF-A	TKF-R
AWGN (Std - 10)	9.9	3.4	3.0	3.1	**2.8**	-
AWGN (Std - 7)	7.1	3.3	2.7	2.9	**2.7**	-
Data Drop-out (50%)	18.2	4.1	4.1	3.4	-	**2.6**
Data Drop-out (25%)	9.5	3.0	3.6	2.8	-	**2.1**
	Real-world D-Mocap Data					
OSU D-Mocap	6.3	6.1	4.4	5.8	**4.1**	-
MHAD D-Mocap	8.4	6.7	5.6	5.3	**4.1**	-

**TABLE 3.** Average bone length error over all frames (cm).

Data Type	Simulation					
	Corrupted Data	Autoencoder Only [23]	TKF Only [61]	KF-A	TKF-A	TKF-R
AWGN (Std - 10)	7.6	1.0	3.3	4.2	**0.8**	-
AWGN (Std - 7)	5.3	1.0	2.6	3.0	**0.8**	-
Data Drop-out (50%)	14.7	1.2	7.7	8.0	-	**1.0**
Data Drop-out (25%)	7.3	1.0	4.6	4.3	-	**0.8**
	Real-world D-Mocap Data					
OSU D-Mocap Data	5.2	0.9	3.7	3.0	**0.8**	-
MHAD D-Mocap Data	2.7	2.5	1.9	2.4	**1.4**	-

**TABLE 4.** Average joint angle error for each dataset (°).

Data Type	Simulation					
	Corrupted Data	Autoencoder Only [23]	TKF Only [61]	KF-A	TKF-A	TKF-R
AWGN (Std - 10)	12.7	3.8	6.4	7.7	**3.3**	-
AWGN (Std - 7)	9.1	3.6	4.6	5.7	**3.3**	-
Data Drop-out (50%)	15.9	4.6	10.6	9.4	-	**2.7**
Data Drop-out (25%)	9.1	3.7	7.6	5.8	-	**2.3**
	Real-world D-Mocap Data					
OSU D-Mocap Data	8.2	4.9	5.3	5.6	**4.2**	-
MHAD D-Mocap Data	11.3	6.9	5.7	6.1	**4.3**	-

**TABLE 5.** Joint angle errors of six joints for each dataset (°).

Data Type	Simulation						
	Joint Angle	Corrupted Data	Autoencoder Only [23]	TKF Only [61]	KF-A	TKF-A	TKF-R
AWGN (Std - 10)	LKF	15.8	4.9	7.0	9.4	**4.0**	-
	LHF	10.8	3.6	6.1	6.8	**3.2**	-
	LHA	12.1	3.1	6.5	7.4	**2.8**	-
	RKF	15.5	4.8	6.9	9.3	**3.8**	-
	RHF	10.3	3.3	6.4	6.4	**3.1**	-
	RHA	11.5	3.2	5.7	7.1	**2.8**	-
	Ave.	12.7	3.8	6.4	7.7	**3.3**	-
AWGN (Std - 7)	LKF	11.3	4.6	4.7	6.9	**3.9**	-
	LHF	7.8	3.4	4.2	5.0	**3.1**	-
	LHA	8.6	3.0	5.1	5.5	**3.0**	-
	RKF	11.2	4.5	4.9	6.8	**4.1**	-
	RHF	7.4	3.2	3.8	4.8	**3.3**	-
	RHA	8.3	3.0	4.8	5.3	**2.7**	-
	Ave.	9.1	3.6	4.6	5.7	**3.3**	-
Data Drop-out (50%)	LKF	21.5	5.5	9.2	13.2	-	**3.5**
	LHF	14.9	4.2	10.8	8.3	-	**3.1**
	LHA	10.9	3.7	11.2	6.5	-	**1.8**
	RKF	22.0	5.9	12.9	13.6	-	**3.2**
	RHF	15.0	4.2	10.3	8.3	-	**2.4**
	RHA	11.2	4.2	9.4	6.7	-	**2.2**
	Ave.	15.9	4.6	10.6	9.4	-	**2.7**
Data Drop-out (25%)	LKF	12.4	4.5	7.5	8.1	-	**2.9**
	LHF	8.2	3.5	7.1	5.0	-	**2.5**
	LHA	6.0	3.2	8.2	4.0	-	**1.6**
	RKF	12.7	4.6	7.6	8.4	-	**2.7**
	RHF	8.7	3.1	6.9	5.0	-	**1.9**
	RHA	6.4	3.4	8.4	4.3	-	**2.0**
	Ave.	9.1	3.7	7.6	5.8	-	**2.3**
	Real-world D-Mocap Data						
OSU D-Mocap Data	LKF	9.8	5.6	5.8	6.0	**4.9**	-
	LHF	8.7	4.9	6.5	5.9	**4.5**	-
	LHA	8.2	4.5	6.3	6.5	**4.2**	-
	RKF	9.4	5.5	4.7	5.9	**4.5**	-
	RHF	3.5	3.3	2.0	3.0	**1.9**	-
	RHA	9.5	5.7	6.5	6.0	**5.0**	-
	Ave.	8.2	4.9	5.3	5.6	**4.2**	-
MHAD D-Mocap Data	LKF	12.8	7.8	6.1	6.7	**4.4**	-
	LHF	11.6	7.2	6.7	6.5	**3.7**	-
	LHA	9.7	5.6	5.7	5.0	**4.7**	-
	RKF	12.8	8.1	5.1	6.8	**4.6**	-
	RHF	11.3	7.0	5.6	6.4	**3.6**	-
	RHA	9.5	5.4	4.9	5.0	**4.8**	-
	Ave.	11.3	6.9	5.7	6.1	**4.3**	-

**TABLE 6.** Body quadrant joint analysis for real-world MHAD D-Mocap.

Joints	Mocap Vel. (cm/s)	D-Mocap Vel. (cm/s)	% Diff. Mocap and D-Mocap	D-Mocap (cm)	Autoencoder [23] (cm)	TKF-A (cm)	TKF-A Improvement (cm)
Upper Body	7.1	31.2	77.2	10.6	8.7	5.5	3.2
Lower Body	2.3	9.1	75.1	6.7	5.1	3.0	2.1
Left Side	4.4	15.1	71.1	8.4	6.8	4.1	2.7
Right Side	4.8	25.9	81.6	9.0	7.2	4.4	2.8
Upper Left	7.1	23.7	70.0	10.4	8.6	5.4	3.2
Upper Right	8.0	45.8	82.5	11.7	9.8	6.1	3.7
Lower Left	2.3	8.7	73.4	6.9	5.4	3.1	2.3
Lower Right	2.3	10.1	79.0	7.0	5.1	3.1	2.0

**TABLE 7.** Run-time comparison.

Method	Online Methods		
	Run-time (sec/frame)	Hardware	Software
EKF [49]	real-time	-	-
TKF [37]	real-time	-	-
LGP [35]	real-time	Intel core 2 duo 3.17 GHz	-
GP [14]	real-time	Intel I7–4720HQ	-
Autoencoder [23]	real-time	Quadro K2000 GPU	Python, Theano
BRA [34]	0.000723	Intel Xeon E5–1620 CPU	-
		Nvidia GTX 1050 Ti GPU	
	Offline Methods		
Sparse Coding [58]	Pose - 1.72	Intel Xeon x5650 2.66 GHz	MATLAB
	Poselet - 0.58		
Sparse Coding [19]	Pose - 0.073	-	-
	Poselet - 0.037		
TKF-assisted Autoencoder (Ours)	0.18	Intel Core i7-8700K 3.7 GHz	Python, Theano, MATLAB
		Nvidia GTX 1080	
		Intel Core i7-4770	

## Footnotes

1
https://github.com/natelannan-osu/MHAD-D-Mocap

2 The OSU dataset has an active IRB Approval from OSU (EN-18-8).
